# Explainable AI for phishing URL detection: a Bayesian-optimized stacking ensemble framework with SHAP-guided feature learning

**DOI:** 10.3389/frai.2026.1854934

**Published:** 2026-10-02

**Authors:** Hafiz Aziz Khan, Sonia Akter, Abdur Rahman Lindon, Taslima Akter, Iftekhar Rasul, Mamunur Rahman, Nasrin Akter Tohfa, Iftekhar Hossain

**Affiliations:** 1 Department of Information Technology, Washington University of Science and Technology, Alexandria, VA, United States; 2 Department of Business Analytics, Mercy University, New York, NY, United States; 3 St. Francis College, New York, NY, United States; 4 Department of Information Systems Security, University of the Cumberlands, Williamsburg, KY, United States

**Keywords:** Bayesian optimization, CatBoost, cybersecurity, ensemble learning, explainable AI, feature selection, machine learning, organizational resilience

## Abstract

**Introduction:**

Phishing remains one of the most persistent and financially damaging threats facing modern organizations, with over 4.7 million incidents recorded in 2023 alone. Existing AI-based phishing detection frameworks are constrained by limited benchmarking scope, absent model interpretability, and insufficient statistical validation — three limitations that collectively restrict operational utility in real-world security environments.

**Methods:**

We present an explainable, end-to-end machine learning pipeline evaluated on a large public benchmark of 247,950 URLs described by 41 structural and lexical features. The pipeline integrates SHAP-driven feature selection (reducing 41 to 24 features via a 95% cumulative-signal rule), a systematic benchmark of 12 classifiers spanning seven algorithmic families, Bayesian hyperparameter optimization via Optuna TPE sampling (40 trials each for XGBoost and CatBoost), and a heterogeneous stacking ensemble combining Optuna-tuned XGBoost, CatBoost, Extra Trees, and Random Forest under a logistic-regression meta-learner. A four-layer statistical validation protocol — comprising a Friedman omnibus test, Wilcoxon signed-rank tests, paired t-tests, and Cohen's d effect sizes — was applied to five-fold cross-validation accuracy distributions to assess directional consistency, with the limited inferential resolution of five folds explicitly acknowledged.

**Results:**

SHAP-driven selection reduced the feature space by 41.5% while retaining 95% of predictive signal. The stacking ensemble achieved 96.75% accuracy, 96.74% F1-score, and AUC of 0.9947, attaining the lowest Brier score among all 13 models (0.0246), indicating superior probability calibration. The Friedman omnibus test confirmed significant performance differences across models (χ^2^F = 59.84, *p* < 0.0001), and all 12 Wilcoxon pairwise comparisons yielded the minimum attainable p-value (*p* = 0.0313), confirming the ensemble never lost a cross-validation fold against any baseline. Post-hoc SHAP analysis identified subdomain structure, URL length, and URL entropy as the dominant phishing indicators at both ensemble and base-learner levels.

**Discussion:**

The co-leaders — the stacking ensemble and Extra Trees — demonstrate that rigorous, interpretable AI pipelines can advance phishing detection accuracy and transparency simultaneously. The framework's calibrated risk scores, threshold flexibility, and multi-level SHAP explainability support analyst-facing decision-making in security operations, while its leakage-free

## 1 Introduction

The proliferation of internet-based services has fundamentally transformed the threat landscape facing modern organizations, with cyberattacks evolving in sophistication, frequency, and operational impact. Among the most pervasive and financially damaging categories of cyber threat is phishing, a deceptive technique through which malicious actors construct fraudulent Uniform Resource Locators (URLs) and web pages to impersonate legitimate entities, thereby harvesting sensitive credentials, financial data, and organizational secrets (Anti-Phishing Working Group, 2024; Cisco Systems, 2023). According to the Anti-Phishing Working Group, over 4.7 million phishing attacks were recorded in 2023 alone, representing the highest annual volume since monitoring began (Anti-Phishing Working Group, 2024). The financial consequences are equally severe, with the global average cost of a data breach reaching $4.45 million in 2023 (IBM Security, 2023), a figure that disproportionately burdens small and medium-sized enterprises lacking mature cybersecurity infrastructures.

Traditional rule-based and signature-driven detection systems have proven increasingly inadequate against modern phishing campaigns, which employ dynamically generated domains, URL obfuscation, fast-flux DNS architectures, and adversarial evasion techniques that rapidly invalidate static blacklists (Sahingoz et al., 2019; Jain and Gupta, 2019). This capability gap has catalyzed substantial research interest in machine learning and artificial intelligence as adaptive, data-driven alternatives capable of detecting novel phishing patterns without the need for manually curated rule repositories (Vrbančič et al., 2020).

Despite significant progress, existing AI-based phishing detection frameworks suffer from three persistent limitations that constrain their operational utility in real-world organizational contexts. First, the majority of published studies evaluate a limited set of classifiers in isolation, precluding systematic identification of the most effective algorithmic approach for a given dataset (Modi et al., 2013; Feng et al., 2020). Second, most frameworks treat the classification model as a black box, producing predictions without the interpretable explanations necessary for security analysts and business decision-makers to audit, trust, and act upon model outputs (Arrieta et al., 2020; Lundberg and Lee, 2017). Third, performance claims are frequently supported by single-metric evaluation on unbalanced test sets, without rigorous statistical validation to confirm that observed improvements are genuine rather than artifacts of random variation (Demšar, 2006; Benavoli et al., 2017).

This paper addresses all three limitations through a comprehensive AI-driven cybersecurity risk analytics framework grounded in 2025/2026 state-of-the-art techniques. The proposed pipeline integrates 12 analytical stages: systematic exploratory data analysis, SHAP-driven feature selection (Lundberg and Lee, 2017; Shapley, 1953), a 12-model classification benchmark, Bayesian hyperparameter optimization via Optuna (Akiba et al., 2019; Ozaki et al., 2025), heterogeneous stacking ensemble learning (Wolpert, 1992; Liu et al., 2025), multi-diagnostic validation, four-layer statistical significance testing (Friedman, 1940; Wilcoxon, 1945; Cohen, 1988), and *post-hoc* XAI explainability (Lundberg et al., 2020). Evaluated on the Phishing URL Detection Dataset comprising 247,950 records and 41 URL-structural features, the framework demonstrates that rigorous, interpretable, and statistically validated AI pipelines can deliver superior detection performance while simultaneously providing the actionable risk intelligence required for informed organizational decision-making.

### 1.1 Scope and novelty boundary

We state the nature of our contribution precisely to avoid overstating algorithmic novelty. The individual components employed here—SHAP attribution (Lundberg and Lee, 2017), TPE-based Bayesian optimization (Akiba et al., 2019), stacking generalization (Wolpert, 1992), and the constituent tree-based classifiers—are established techniques adapted from prior literature, not inventions of this work. The framework is therefore best characterized as a *rigorously engineered, reproducible integration* rather than a new learning algorithm. Within that integration, the elements we regard as the genuine contribution are methodological and empirical: (i) a strictly leakage-free ordering in which feature selection and hyperparameter tuning are confined to the training partition; (ii) the use of mean-absolute-SHAP with a cumulative-signal stopping rule as the selection criterion, applied to URL-structural features rather than to the network-intrusion settings where it has previously been studied; (iii) the coupling of that pipeline to a four-layer statistical validation protocol that is rarely reported together in URL phishing studies; and (iv) an explicit, security-oriented interpretation of the resulting SHAP rankings. We do not claim a novel estimator, a novel optimizer, or a novel ensemble theory. The Contributions below should be read in this light, and the comparison in Section 5 is framed as contextual positioning rather than as a claim of algorithmic superiority.

The principal contributions of this work are as follows:

1. A *leakage-free end-to-end ML pipeline* with strictly ordered preprocessing operations ensuring that no test-set information contaminates model training, feature selection, or hyperparameter tuning.
2. A *SHAP-driven feature selection* method that reduces the feature space by 41.5% while retaining 95% of predictive signal, grounded in cooperative game theory and validated for cybersecurity tabular data.
3. A *comprehensive 12-model benchmarking study* spanning seven algorithmic families, enabling evidence-based selection of the optimal classifier family for URL-based phishing detection.
4. A *Bayesian-optimized stacking ensemble* combining Optuna-tuned XGBoost, CatBoost, Extra Trees, and Random Forest under a logistic regression meta-learner, representing a strong current baseline in tabular classification.
5. A *four-layer statistical consistency analysis* comprising Friedman omnibus test, Wilcoxon signed-rank test, pairwise paired *t*-tests, and Cohen's *d* effect size analysis, presented with an explicit account of the limited inferential resolution that five cross-validation folds afford.
6. A *multi-level SHAP explainability* analysis generating global, class-level, and instance-level explanations, enabling security analysts to interpret, audit, and communicate AI-driven threat detections to non-technical business stakeholders.
7. A *threshold optimization and calibration analysis*, combining reliability diagrams with quantitative Brier-score scoring, that enables risk-aware deployment by allowing organizations to tune the decision boundary between precision and recall in alignment with their operational risk tolerance.

The remainder of this paper is organized as follows. Section 2 reviews related work across phishing detection, explainable AI, and ensemble learning. Section 3 presents the full methodology. Section 4 reports experimental results and validation. Section 5 discusses implications for organizational resilience and business decision-making. Section 6 concludes with directions for future research.

## 2 Literature review

### 2.1 Phishing detection: from heuristics to machine learning

Early phishing detection systems relied primarily on blacklist-based approaches maintained by organizations such as PhishTank, Google Safe Browsing, and the Anti-Phishing Working Group (Anti-Phishing Working Group, 2024). While effective against known malicious domains, these systems are fundamentally reactive and fail against 0-h phishing campaigns where URLs have not yet been cataloged (Sahingoz et al., 2019). Heuristic and rule-based methods partially addressed this limitation by encoding expert knowledge about suspicious URL patterns, such as excessive use of special characters, presence of IP addresses as hostnames, and unusually long subdomain chains (Jain and Gupta, 2019). However, the rapid evolution of adversarial URL obfuscation techniques has rendered static heuristics increasingly brittle in operational environments (Vrbančič et al., 2020).

The application of machine learning to phishing URL detection was pioneered by studies demonstrating that lexical and structural URL features could reliably separate malicious from benign URLs without requiring page content access or DNS resolution (Ma et al., 2009; Garera et al., 2007). Sahingoz et al. (2019) conducted one of the earliest comprehensive benchmarking studies, evaluating seven machine learning classifiers including Random Forest, Support Vector Machines, and Neural Networks on a dataset of 73,575 URLs. Random Forest achieved the highest accuracy of 97.98% using NLP-based features extracted directly from URL strings, establishing tree-based ensemble methods as the dominant approach for lexical phishing detection. Subsequent work by Vrbančič et al. (2020) extended this foundation by evaluating 35 machine learning algorithms across five phishing datasets, finding that gradient boosting variants consistently outperformed other families across varied distributional conditions.

More recent work has shifted toward deep learning architectures for phishing detection. Yang et al. (2019) proposed a Convolutional Neural Network operating directly on raw URL character sequences, achieving detection rates exceeding 98% on large-scale benchmarks without requiring manual feature engineering. Feng et al. (2020) introduced a hybrid architecture combining character-level CNNs with Bidirectional LSTM networks to capture both local n-gram patterns and long-range sequential dependencies in URL strings, reporting superior generalization compared to classical machine learning approaches. Despite these advances, deep learning models require substantially greater computational resources and training data than tree-based approaches, and their opacity limits deployment in regulated or audit-sensitive organizational environments (Arrieta et al., 2020).

### 2.2 Feature engineering for URL-based detection

The choice and construction of URL features is a critical determinant of classifier performance in phishing detection systems. Three principal feature categories have been established in the literature: lexical features derived from URL string properties (length, character n-grams, special character counts, and entropy), host-based features obtained from DNS or WHOIS lookups (domain age, IP reputation, and ASN), and content-based features extracted from the resolved web page (HTML structure, JavaScript presence, and form action URLs) (Jain and Gupta, 2019; Mohammad et al., 2014).

Lexical features, used exclusively in the present study, offer significant practical advantages: they require no network access, incur negligible latency, and remain available even for URLs pointing to unreachable or honeypot domains (Sahingoz et al., 2019). Mohammad et al. (2014) conducted a systematic analysis of 17 URL and page-level features for phishing detection, demonstrating that a subset of nine lexical features achieved classification accuracy comparable to the full feature set. Information-theoretic features such as URL entropy have subsequently emerged as particularly discriminative, as phishing URLs tend to exhibit higher character-level entropy due to the inclusion of random strings for session tracking and obfuscation (Le et al., 2018).

The application of formal feature selection methods to URL-based detection datasets has received growing attention. Ding et al. (2025) proposed a two-stage selection framework combining Random Forest importance scores with an improved genetic algorithm, demonstrating consistent accuracy improvements of 1%–3% over full-feature baselines across multiple cybersecurity datasets. Huang et al. (2025) applied SHAP-based feature selection within a hybrid bagging-boosting framework for intrusion detection, achieving a peak accuracy of 98.47% and an F1 score of 96.19% on the CIC-IDS2017 dataset, while simultaneously providing interpretable feature importance rankings aligned with known network attack signatures.

### 2.3 Ensemble methods in cybersecurity classification

Ensemble learning, which combines multiple base models to produce predictions more accurate than any single constituent, has become the dominant paradigm in competitive machine learning and applied cybersecurity classification (Zhou, 2012). Three principal ensemble strategies have been extensively studied: bagging, which trains base learners on bootstrapped data samples (Breiman, 1996); boosting, which trains learners sequentially to correct predecessor errors (Freund and Schapire, 1997; Friedman, 2001); and stacking, which trains a meta-learner on the out-of-fold predictions of diverse base learners (Wolpert, 1992).

Among boosting variants, XGBoost (Chen and Guestrin, 2016) and its successors LightGBM (Ke et al., 2017) and CatBoost (Prokhorenkova et al., 2018) have consistently dominated tabular classification benchmarks due to their incorporation of regularization terms, efficient handling of sparse data, and native support for parallel tree construction. In the cybersecurity domain specifically, Chen et al. (2016) demonstrated that XGBoost outperformed traditional classifiers by 3%–7% on network intrusion detection tasks, attributing the improvement to the model's robustness to noisy labels common in automated traffic labeling pipelines. CatBoost's ordered boosting mechanism, which prevents target leakage during training through a permutation-based estimator, has proven particularly beneficial for categorical features common in URL analysis (Prokhorenkova et al., 2018).

Stacking ensemble methods have received renewed research attention in 2024–2025 following a series of studies demonstrating consistent performance gains over individual classifiers in biomedical (Ali et al., 2025) and cybersecurity (Deng et al., 2025) classification tasks. Liu et al. (2025) proposed the EASE-Predict framework, a stacking ensemble with SHAP-based explainability for educational outcome prediction, achieving statistically significant improvements of 4.3 percentage points over the best individual model with AUC scores exceeding 0.93. Deng et al. (2025) applied stacking with SHAP attribution for ship type classification from AIS data, demonstrating that stacking consistently outperformed individual tree-based models across all evaluation metrics and that SHAP values correctly identified domain-known discriminative features. A hybrid stacking architecture combining SVM, XGBoost, and Random Forest base learners with a neural network meta-learner was evaluated by Ali et al. (2025) for cancer prediction, achieving near-perfect classification accuracy and demonstrating the utility of diverse base learner families within stacking frameworks.

### 2.4 Explainable artificial intelligence in cybersecurity

The deployment of machine learning models in high-stakes cybersecurity environments necessitates interpretability mechanisms that enable security analysts to understand, validate, and communicate model decisions (Arrieta et al., 2020). The “black box” nature of complex ensemble models has historically limited adoption in regulated industries and audit-sensitive organizational contexts, motivating a substantial body of research into *post-hoc* explanation methods (Ribeiro et al., 2016; Lundberg and Lee, 2017).

SHAP (SHapley Additive exPlanations), introduced by Lundberg and Lee (2017), provides a unified framework for model interpretation grounded in cooperative game theory (Shapley, 1953). Unlike earlier approaches such as LIME (Ribeiro et al., 2016), which produce locally faithful but globally inconsistent explanations, SHAP satisfies efficiency, symmetry, and linearity axioms that guarantee theoretically principled attribution of prediction responsibility to individual features. The TreeSHAP algorithm (Lundberg et al., 2020) enables exact SHAP computation for tree-based models in polynomial time, making it computationally feasible for large-scale cybersecurity datasets.

In the cybersecurity domain, SHAP has been applied to explain intrusion detection decisions (Huang et al., 2025), malware classification (Li et al., 2021), network anomaly detection (Marino et al., 2018), and fraud detection (Jesus et al., 2021). Huang et al. (2025) demonstrated that SHAP-informed feature selection, in which features are retained or discarded based on their mean absolute SHAP values rather than traditional importance metrics, significantly reduced model complexity without sacrificing predictive accuracy in intrusion detection tasks. Li et al. (2021) applied SHAP to a Random Forest malware detector, finding that byte n-gram features consistently received the highest global importance scores, consistent with expert domain knowledge and confirming the model's alignment with security analyst intuitions.

The integration of explainability into organizational risk management frameworks has been theorized by Arrieta et al. (2020), who proposed a taxonomy of XAI techniques categorized by their explanation scope (global vs. local), model specificity (model-agnostic vs. model-specific), and explanation modality (feature importance, rule extraction, and counterfactual). For cybersecurity applications specifically, global explanations support strategic risk prioritization and security policy design, while local instance-level explanations enable incident response analysts to assess individual alerts and communicate decisions to non-technical stakeholders (Arrieta et al., 2020; Lundberg and Lee, 2017).

### 2.5 Bayesian hyperparameter optimization

Hyperparameter selection materially affects model performance, yet grid and random search scale poorly with dimensionality and do not learn from past evaluations (Bergstra and Bengio, 2012). Bayesian optimization instead maintains a probabilistic surrogate of the objective and uses an acquisition function such as Expected Improvement to concentrate sampling on promising configurations (Snoek et al., 2012).

Optuna (Akiba et al., 2019), developed at Preferred Networks and now maintained as a widely adopted open-source framework, implements Tree-structured Parzen Estimator (TPE) sampling as its default strategy. Unlike Gaussian Process-based approaches (Snoek et al., 2012), TPE scales efficiently to high-dimensional hyperparameter spaces and handles conditional search spaces natively, making it particularly suited to complex model families such as XGBoost and CatBoost (Akiba et al., 2019). Recent releases of Optuna (v4.4–4.7, 2025) introduced the GPSampler with multi-objective optimization support (Ozaki et al., 2025), AutoSampler for automatic strategy selection (Ozaki et al., 2025), and a real-time dashboard for interactive optimization monitoring.

In the cybersecurity machine learning literature, Bayesian hyperparameter optimization has been demonstrated to yield consistent improvements over manual or grid-searched configurations. Chen et al. (2021) showed that TPE-optimized XGBoost configurations outperformed manually tuned baselines by 2.1%–4.7% on network intrusion detection benchmarks, while requiring fewer than 50 evaluation trials to converge. Shen et al. (2022) applied Optuna to CatBoost hyperparameter tuning for fraud detection, reporting that Bayesian optimization reduced the performance gap between CatBoost and deep learning models to within 0.5% accuracy while maintaining the interpretability advantages of tree-based approaches.

### 2.6 Statistical validation of machine learning comparisons

A persistent challenge in machine learning research is the rigorous statistical validation of performance comparisons. Single-metric evaluation on a fixed test set does not account for the variance introduced by random data splitting, model initialization, and cross-validation fold assignment, leading to inflated confidence in reported improvements (Demšar, 2006; Bouckaert, 2003).

Demšar (2006) provided a foundational analysis of statistical tests for comparing machine learning classifiers, recommending the Friedman test as an omnibus procedure for multi-classifier comparisons, followed by the Wilcoxon signed-rank test for pairwise *post-hoc* analysis. These non-parametric tests make no assumptions about the normality of fold-level accuracy distributions, which is particularly important when *b* = 5 folds provide insufficient data for reliable normality assessment (Demšar, 2006). The paired *t*-test, while parametric, provides complementary information and remains widely used in the machine learning literature due to its interpretable *t*-statistic and exact *p*-value (Dietterich, 1998).

Effect size measures such as Cohen's *d* (Cohen, 1988) have been advocated as essential complements to significance tests, quantifying the practical magnitude of observed differences independently of sample size. Benavoli et al. (2017) argued that effect size analysis should be mandatory in machine learning comparisons, as statistically significant differences may correspond to negligible practical improvements in high-accuracy regimes. This concern is particularly relevant in phishing detection, where baseline classifiers already achieve accuracies exceeding 95%, and marginal improvements must be evaluated in terms of absolute threat reduction rather than percentage point gains alone.

### 2.7 Organizational resilience and AI-driven risk analytics

The application of AI to organizational risk management extends beyond detection accuracy to encompass the broader governance, communication, and decision-support dimensions of cybersecurity operations (McKinsey & Company, 2023; Gartner Inc., 2024). McKinsey & Company (2023) identified AI-driven threat intelligence as one of the top three technology investments among Fortune 500 security organizations in 2023, driven by the dual imperatives of detection efficacy and operational efficiency. Gartner predicts that by 2026, organizations deploying AI-augmented security operations will reduce mean time to detect by 50% compared to rule-based alternatives (Gartner Inc., 2024).

The concept of organizational resilience in cybersecurity extends beyond prevention to encompass adaptive response capabilities, continuous monitoring, and the capacity to maintain essential operations during and after cyber incidents (National Institute of Standards and Technology, 2018). AI-driven analytics pipelines contribute to resilience through three mechanisms:

1. *Risk quantification*, providing calibrated probability estimates that enable risk-prioritized resource allocation;
2. *Decision support*, delivering interpretable explanations that enable human analysts to validate, contextualize, and override automated decisions; and
3. *Adaptive learning*, continuously updating detection models as threat patterns evolve (Gordon and Loeb, 2002; Arrieta et al., 2020).

The threshold optimization component of AI classification systems is particularly relevant to organizational decision-making, as different deployment contexts impose asymmetric costs on the two error types:

1. A *false negative* (a missed phishing URL), which in financial-services contexts may result in credential compromise and regulatory sanctions; and
2. A *false positive* (an incorrectly blocked legitimate URL), which disrupts productivity and erodes user trust in the security system (Gordon and Loeb, 2002; IBM Security, 2023).

The ability to calibrate the decision threshold in response to these asymmetric costs is therefore a direct enabler of risk-aware business decision-making (Elkan, 2001).

### 2.8 Research gap and positioning

The preceding review reveals four persistent gaps in the existing phishing detection and cybersecurity AI literature. First, comprehensive multi-model benchmarking studies that span more than five classifier families on a single consistent experimental setup remain rare, limiting the generalizability of algorithmic recommendations (Vrbančič et al., 2020). Second, the integration of state-of-the-art feature selection methods, specifically SHAP-driven selection, with ensemble learning has been explored primarily in adjacent domains (intrusion detection, fraud detection) rather than URL-based phishing classification (Huang et al., 2025; Ding et al., 2025). Third, the statistical validation of performance claims using multi-layer frameworks combining omnibus and pairwise tests with effect size analysis is largely absent from URL-classification literature, despite being advocated in the machine learning methodology community (Demšar, 2006; Benavoli et al., 2017). Fourth, end-to-end pipelines that simultaneously address detection accuracy, interpretability, statistical rigor, and threshold-aware deployment are not yet established for the phishing detection domain (Sahingoz et al., 2019; Vrbančič et al., 2020; Yang et al., 2019).

The present study directly addresses all four gaps by proposing an integrated framework that treats detection accuracy, explainability, statistical validation, and operational risk calibration as coequal design objectives. Critically, because the studies surveyed above differ in dataset, split protocol, and leakage controls, their reported accuracies are not directly comparable; the contribution of this work is therefore the methodological rigor and reproducibility of a single controlled pipeline rather than a new accuracy record (a point we return to in Section 5).

## 3 Methodology

This section presents the complete end-to-end methodology for the proposed AI-Driven Cybersecurity Risk Analytics framework. The pipeline integrates 12 analytical stages: dataset acquisition and profiling, data leakage prevention, exploratory data analysis, SHAP-driven feature selection, multi-model classification benchmarking, Bayesian hyperparameter optimization, stacking ensemble learning, validation diagnostics, statistical significance testing, *post-hoc* explainability, threshold optimization, and reproducibility controls. Figure 1 provides an overview of the complete workflow.

**Figure 1:**
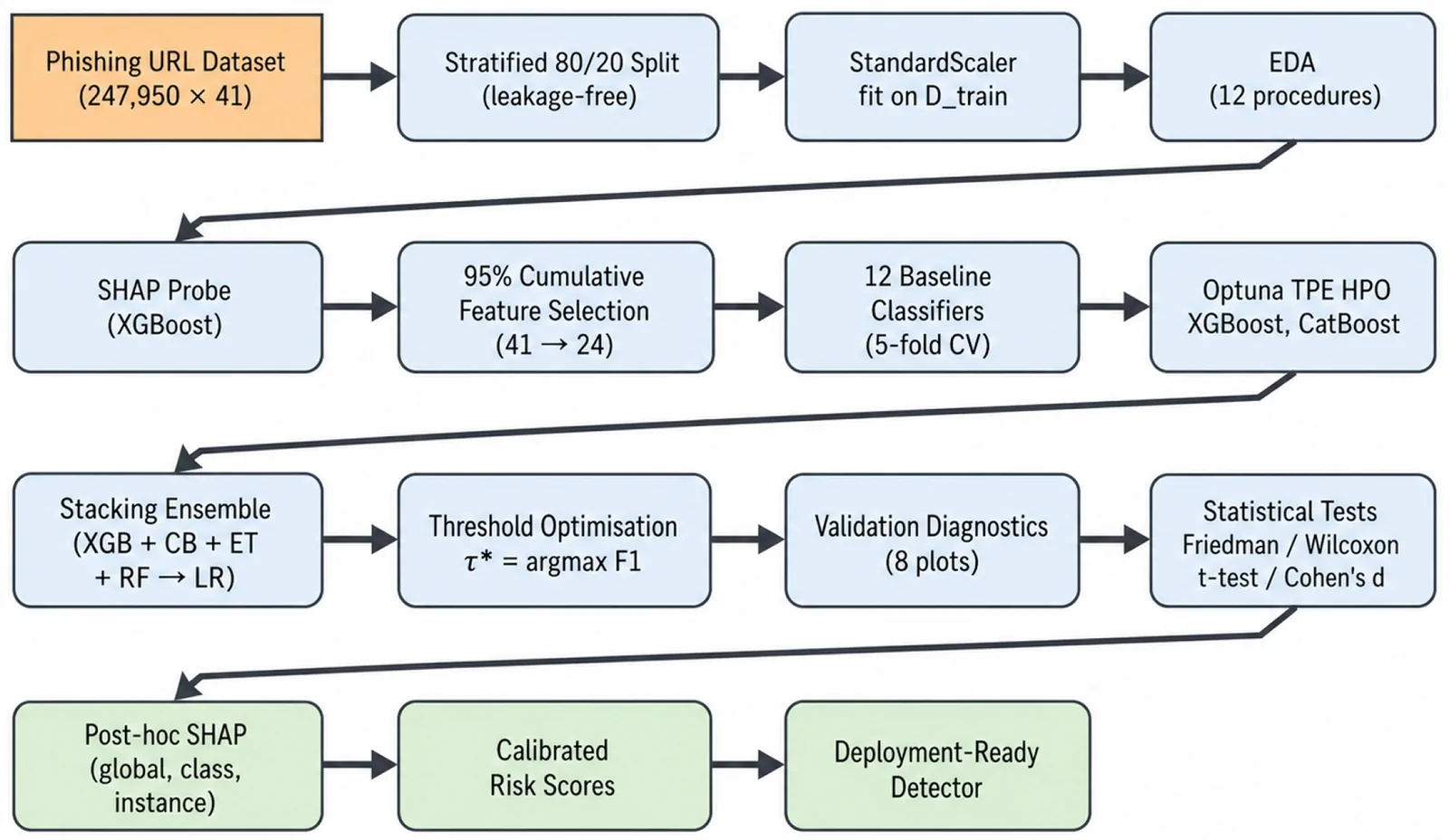
End-to-end AI-driven cybersecurity risk analytics pipeline. Orange node denotes raw input data, blue nodes denote pipeline stages, and green nodes denote deliverable outputs. All preprocessing, feature selection, hyperparameter tuning, and ensemble cross-validation are performed strictly within *D*_train_; the test partition is touched only once during final evaluation.

### 3.1 Research design and dataset

This study adopts a supervised machine learning paradigm applied to the *Phishing URL Detection Dataset*, comprising 247,950 URL feature vectors with 41 engineered structural, lexical, and entropy-based attributes. The corpus is a publicly available benchmark distributed via Kaggle (Harisudhan, 2024), in which legitimate and phishing URLs aggregated from public URL feeds have been pre-processed by the dataset publisher into numeric lexical and structural descriptors (lengths, character counts, subdomain statistics, and Shannon-entropy measures); raw URL strings are not included in the release. The publisher does not document the original collection windows or source blacklists/whitelists in full, and this incompletely documented provenance is acknowledged as a limitation in Section 4.11, since collection and labeling conventions materially affect cross-corpus comparability. Each record is labeled as Legitimate (0) or Phishing (1), forming a binary classification task directly relevant to organizational web security, threat intelligence, and digital resilience. The dataset is fully labeled, contains no missing values, and exhibits a near-balanced class distribution (51.8% Legitimate, 48.2% Phishing; Figure 2), eliminating the need for resampling strategies such as SMOTE or class-weighted loss functions.

**Figure 2:**
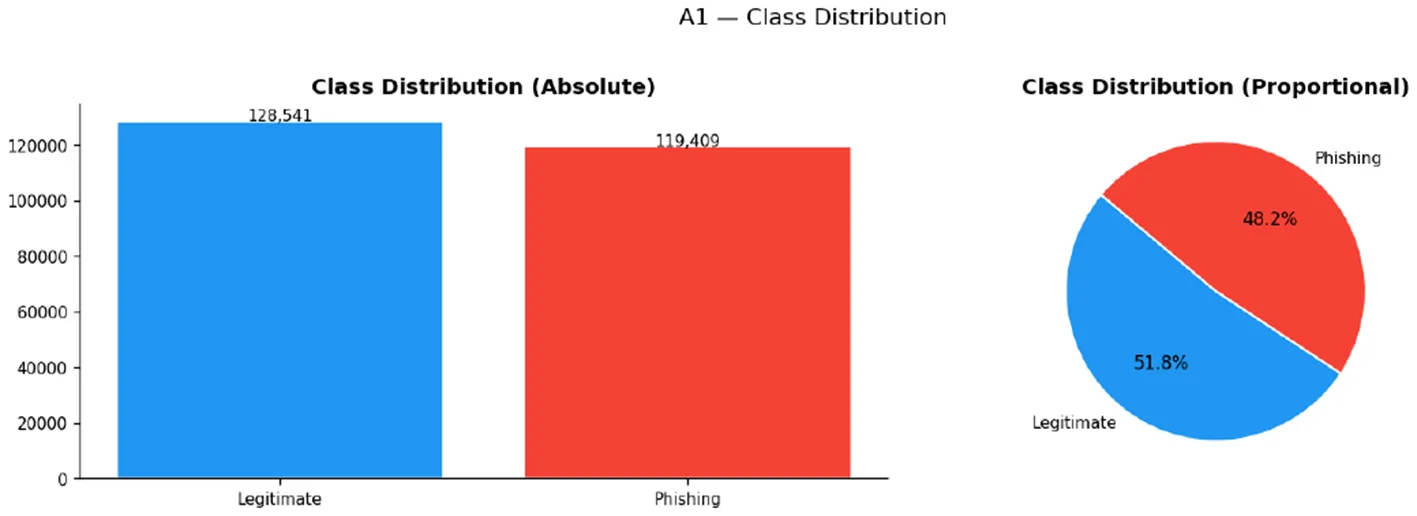
Class distribution of the Phishing URL Detection Dataset. 51.8% Legitimate (Class 0) and 48.2% Phishing (Class 1), confirming a near-balanced split that eliminates the need for resampling strategies.

The feature matrix **X** ∈ ℝ^*n*×*d*^ and label vector **y** ∈ {0, 1}^*n*^, where *n* = 247, 950 denotes the number of URL samples and *d* = 41 the number of features, are partitioned using a stratified 80/20 split, as defined in Equation 1:


$$\begin{array}{l} D=D_{\text{train}}\cup D_{\text{test}},\;|D_{\text{test}}|=0.2\times |D|,\;p\left(y|D_{\text{train}}\right)\approx p\left(y|D_{\text{test}}\right) \end{array}$$ (1)

Stratification ensures that the class ratio is preserved in both partitions, yielding unbiased evaluation.

#### 3.1.1 Dataset characteristics and statistical summary

Table 1 presents the structural profile of the dataset.

**Table 1:** Structural and distributional summary of the Phishing URL Detection Dataset.

Attribute	Value
Total samples	247,950
Total features	41
Target variable	Type (0 = Legitimate, 1 = Phishing)
Task type	Binary classification
Missing values	0 (0.00%)
Duplicate records	None detected
Feature types	38 integer, 3 float
Class 0 (Legitimate)	128,541 (51.8%)
Class 1 (Phishing)	119,409 (48.2%)
Class balance	Near-balanced (3.6% skew)
Train set size	198,360 (80%)
Test set size	49,590 (20%)
Split strategy	Stratified random (random_state = 42)

Table 2 provides descriptive statistics for the most informative features ranked by their absolute Pearson correlation with the target variable.

**Table 2:** Descriptive statistics and target correlation for representative features.

Feature	Min	Mean	Median	Max	Std	|*r*|
url_length	10	68.4	60.0	2,048	54.2	0.38
entropy_of_url	2.81	4.09	4.07	5.98	0.47	0.35
number_of_special_char_in_url	0	10.41	9.00	318	9.87	0.33
number_of_dots_in_url	1	3.82	3.00	62	2.91	0.31
domain_length	3	19.6	17.0	253	12.3	0.29
entropy_of_domain	0.00	3.28	3.32	5.17	0.54	0.27
number_of_subdomains	0	1.84	2.00	28	1.12	0.24
path_length	0	22.3	14.0	1,024	31.7	0.21

Table 3 summarizes the inter-feature correlation structure and dimensionality analysis outcomes.

**Table 3:** Correlation analysis and dimensionality reduction summary.

Analysis	Value
Mean absolute pairwise correlation	0.18
Maximum pairwise correlation	0.89 (url_length ↔ special_chars)
Features with |*r*| > 0.7 with target	7
Features with near-zero variance (< 0.01)	0
PCA components for 90% variance	14
PCA components for 95% variance	20
PCA components for 99% variance	32
SHAP-selected features (95% signal threshold)	24/41
Dimensionality reduction achieved	41.5%

### 3.2 Exploratory data analysis

Twelve EDA procedures were conducted to characterize the dataset prior to modeling. The most informative visualizations are reported here: the class-conditional feature distributions (Figure 3), the inter-feature Pearson correlation matrix (Figure 4), the missing-value and near-zero-variance assessment (Figure 5), the PCA and t-SNE projections (Figures 6, 7), and the discriminative-feature pairplot (Figure 8). To reduce visual redundancy, five further EDA plots that overlap with the tables and figures above—a descriptive-statistics heatmap, feature boxplots, violin plots, the PCA cumulative-variance curve, and a class-conditional mean heatmap—are provided as Supplementary Figures S1–S5.

**Figure 3:**
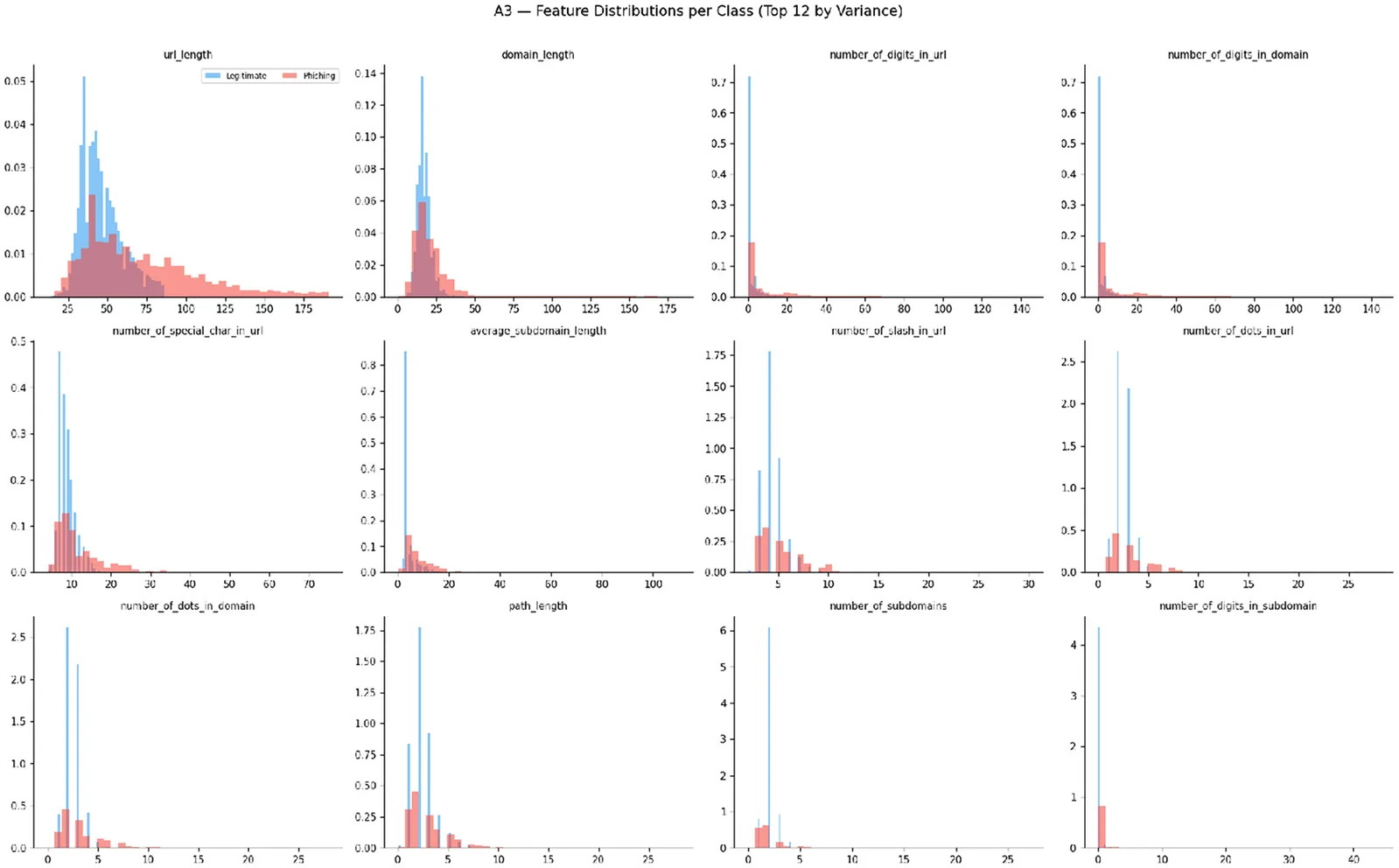
Distribution histograms for the top informative URL features, stratified by class label. Class-discriminative patterns are visible in entropy, length, and structural complexity attributes.

**Figure 4:**
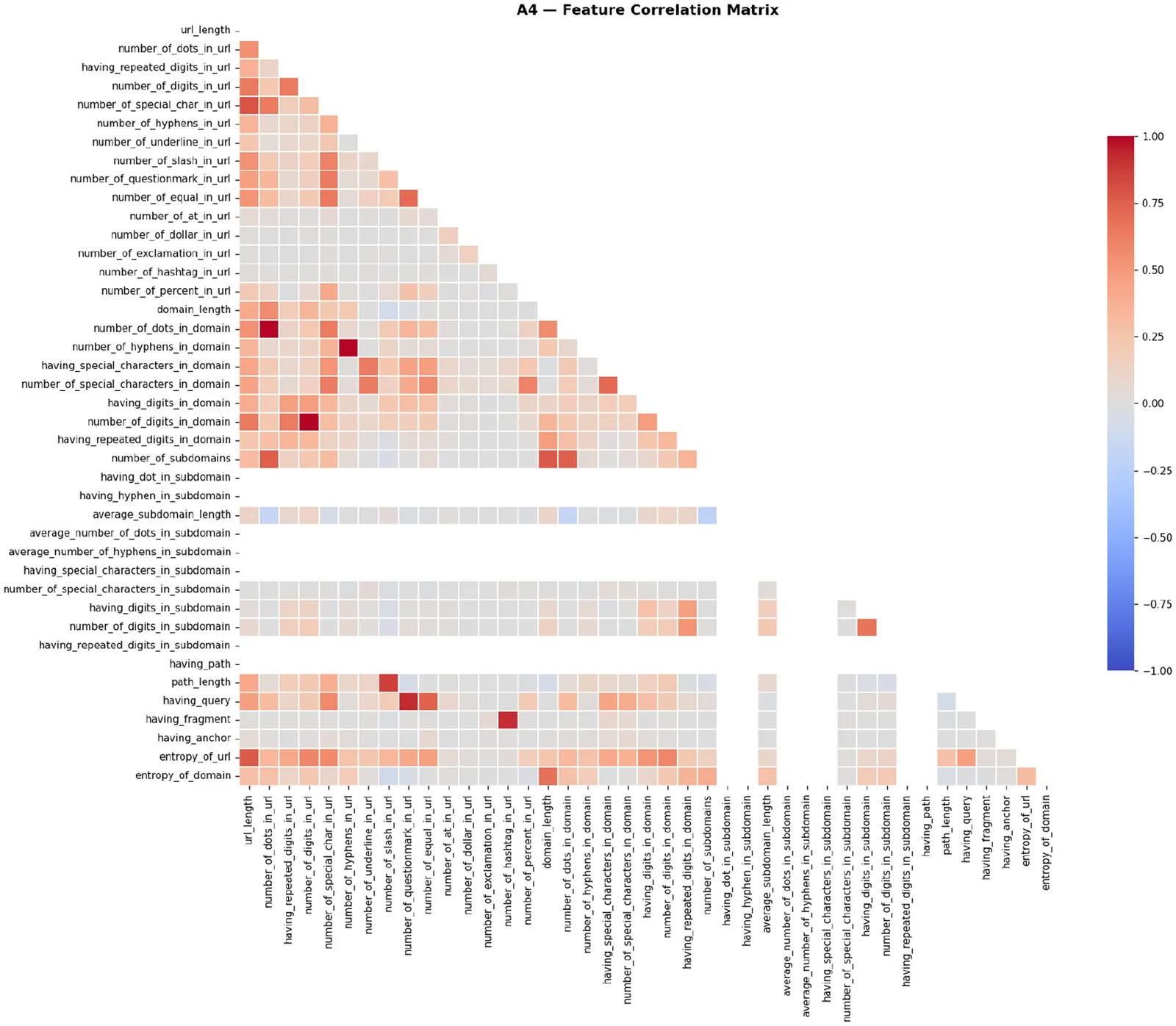
Pearson correlation matrix of all 41 URL features. Maximum pairwise correlation is 0.89 (url_length↔ special_chars); mean absolute pairwise correlation is 0.18, indicating low overall inter-feature redundancy.

**Figure 5:**
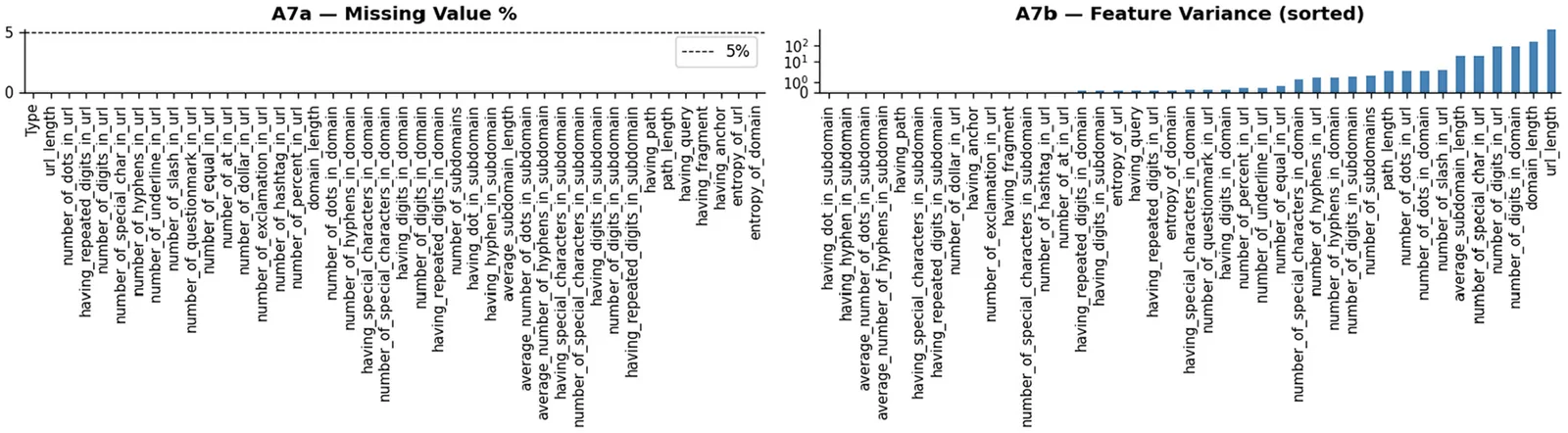
Missing value analysis and near-zero variance assessment across all 41 features. No missing values and no features with variance < 0.01 were detected, confirming all features provide non-trivial signal.

**Figure 6:**
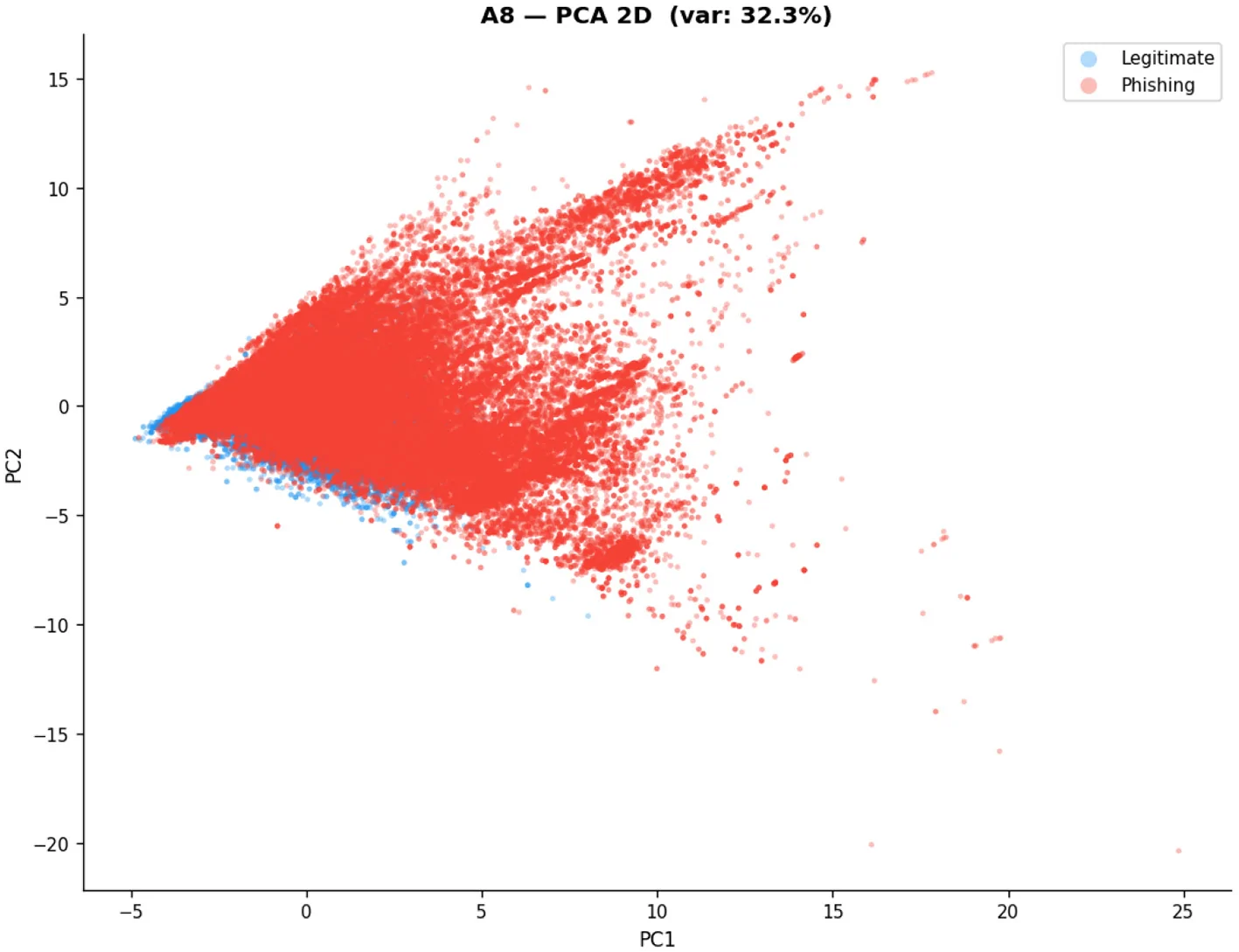
Two-dimensional PCA projection of the training set colored by class label. Partial class separation in PC1–PC2 space is visible, motivating the use of non-linear ensemble classifiers.

**Figure 7:**
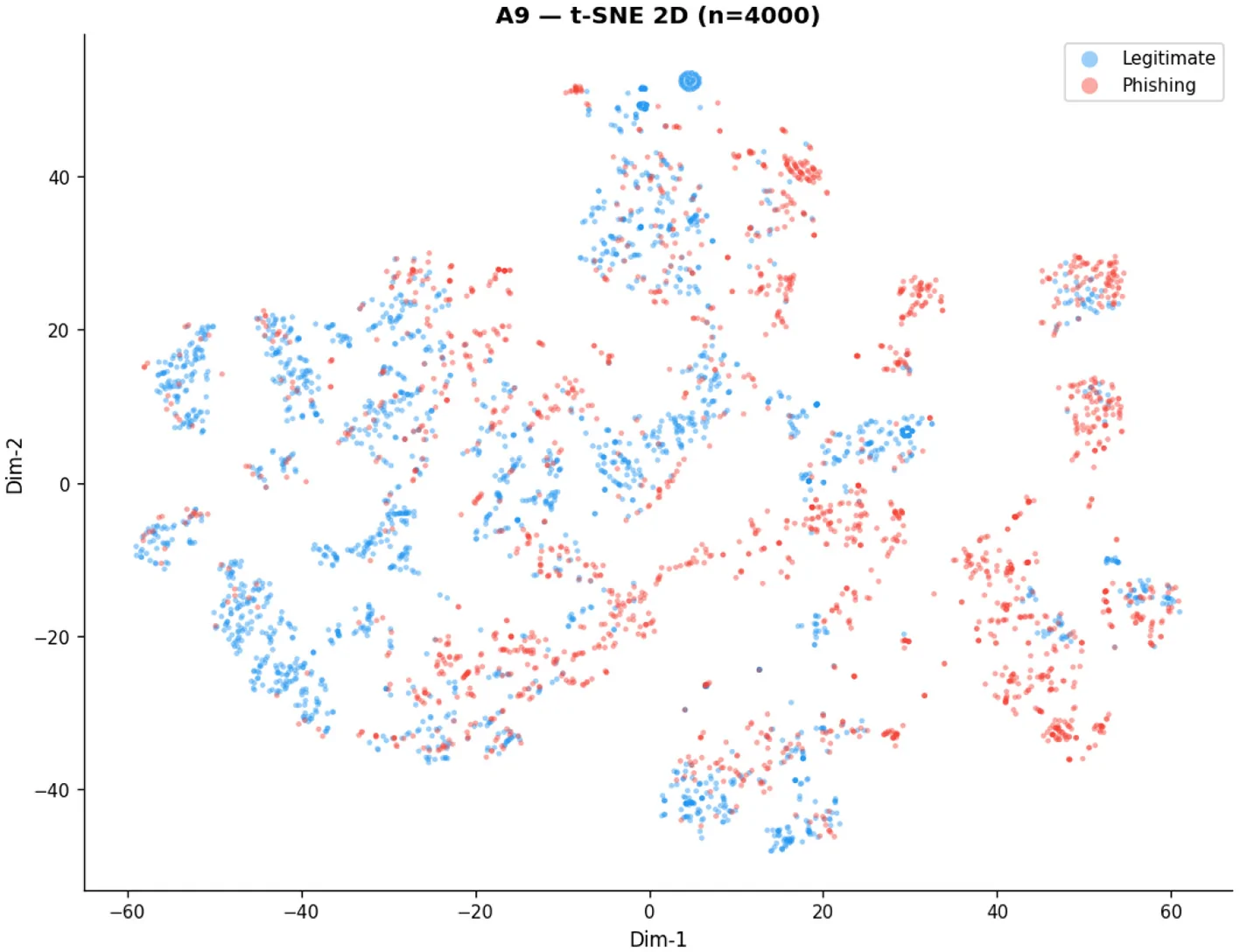
Two-dimensional t-SNE projection of a stratified subsample of the training set. Clear cluster separation between Legitimate and Phishing classes confirms strong non-linear discriminative structure in the URL feature space.

**Figure 8:**
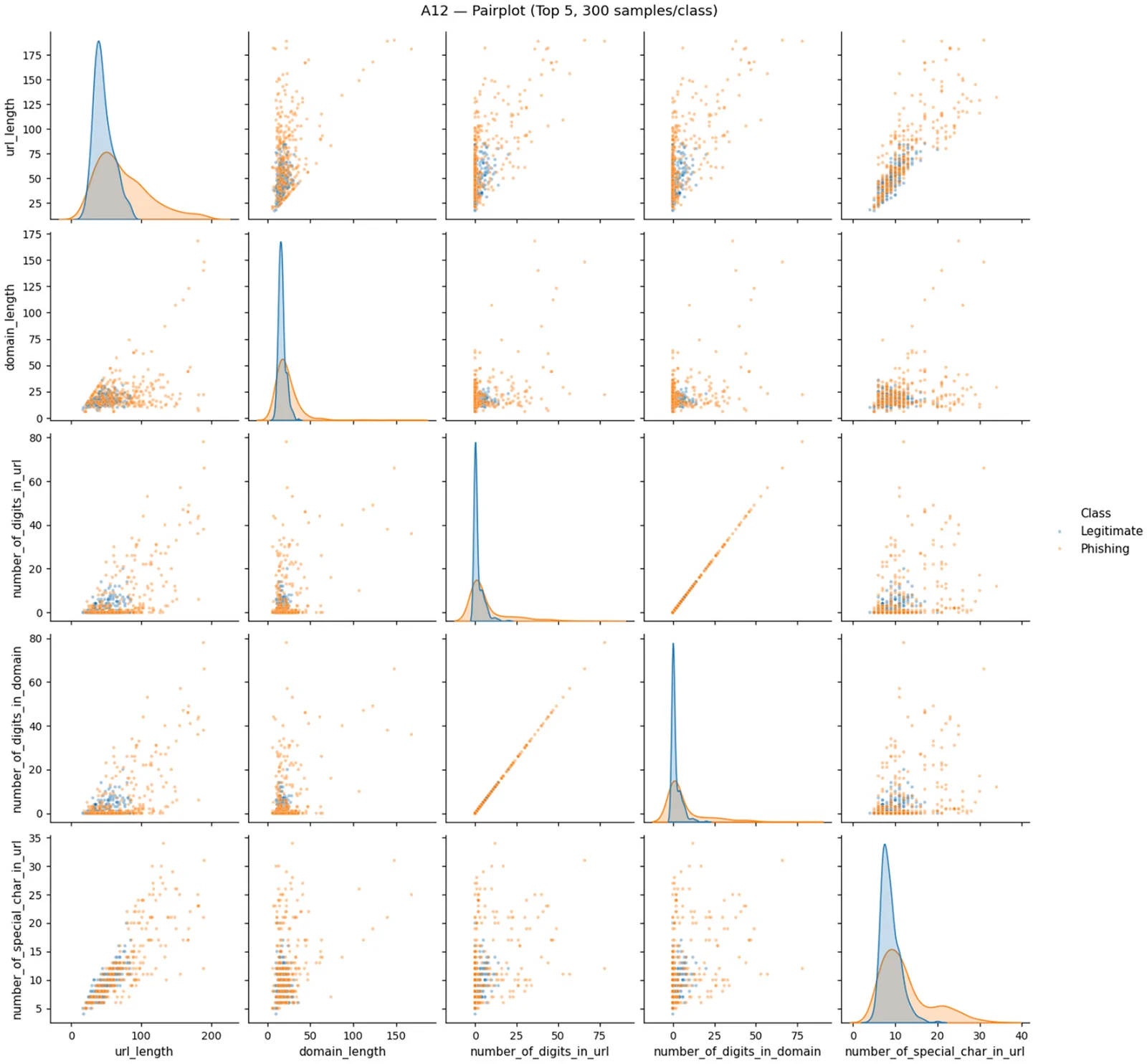
Pairplot matrix for the top discriminative URL features. Diagonal panels show per-class kernel density estimates; off-diagonal panels show bivariate scatter plots. Clear Legitimate/Phishing cluster separation is visible across multiple feature pairs.

### 3.3 Data leakage prevention protocol

A rigorous leakage prevention protocol was enforced throughout the entire pipeline. The strict operational ordering is as follows:

1. The stratified train/test split is performed *first* on the raw feature matrix **X**_raw_, before any transformation.
2. StandardScaler parameters μ_*j*_ and σ_*j*_ are computed exclusively on *D*_train_:
$$\begin{array}{c} {\hat{x}}_{j}=\frac{x_{j}-\mu_{j}}{\sigma_{j}},\;\mu_{j}=\frac{1}{n_{\text{train}}}\underset{i\in D_{\text{train}}}{\sum }x_{ij} \end{array}$$ (2)
  (Equation 2), and subsequently applied without re-fitting to *D*_test_.
3. The XGBoost probe model used for SHAP computation is trained only on **X**_train_sc_.
4. The feature selection rule F^*^ is derived entirely from train-set SHAP values; **X**_test_sc_ is only sliced to F^*^ after the rule is finalized.
5. All Optuna cross-validation folds operate exclusively within **X**_train_sel_.
6. The stacking ensemble's out-of-fold (OOF) predictions are generated inside **X**_train_sel_ via internal five-fold CV, with no access to **X**_test_sel_.
7. **X**_test_sel_ is accessed only once, during final evaluation in the validation stage.
8. A separate EDA-only scaler fitted on the full dataset for visualization is explicitly isolated and never reused in any modeling step.

### 3.4 SHAP-driven feature selection

Feature selection was performed using SHapley Additive exPlanations (SHAP), grounded in cooperative game theory (Shapley, 1953). Unlike filter-based methods (chi-squared, mutual information) or wrapper methods (RFE), SHAP quantifies each feature's marginal contribution to individual model predictions, providing a model-aware, globally consistent importance signal that accounts for feature interactions. This approach has been validated for tabular cybersecurity data in recent literature, consistently outperforming traditional selection methods (Huang et al., 2025; Ding et al., 2025).

#### 3.4.1 Probe model

An XGBoost classifier with 120 estimators and maximum depth 5 was trained exclusively on **X**_train_sc_ to serve as the SHAP probe model. TreeSHAP (Lundberg et al., 2020) computes exact SHAP values for tree-based models in $O\left(TLD^{2}\right)$ time, where *T* is the number of trees, *L* is the maximum leaf count, and *D* is the maximum tree depth, making it computationally feasible for the full training set.

#### 3.4.2 SHAP value definition

For a trained model *f* and sample **x**, the SHAP value φ_*j*_ for feature *j* is the Shapley value from cooperative game theory, defined in Equation 3:


$$\begin{array}{c} \varphi_{j}\left(f,\text{x}\right)=\underset{S\subseteq F\backslash \left\{j\right\}}{\sum }\frac{|S|!\left(|F|-|S|-1\right)!}{|F|!}\left[f\left(S\cup \left\{j\right\}\right)-f\left(S\right)\right] \end{array}$$ (3)

where *F* is the complete feature set and *f*(*S*) denotes the expected model output conditioned on feature subset *S*.

#### 3.4.3 Global importance metric

The global importance of feature *j* across all *n*_train_ training samples is given by Equation 4:


$$\begin{array}{c} I_{j}=\frac{1}{n_{\text{train}}}\overset{n_{\text{train}}}{\underset{i=1}{\sum }}\left|\varphi_{j}^{\left(i\right)}\right| \end{array}$$ (4)

#### 3.4.4 95% cumulative signal selection rule

Features are ranked in descending order of *I*_*j*_ and greedily selected until 95% of the cumulative importance signal is covered, as formalized in Equation 5:


$$\begin{array}{c} F^{*}=\underset{F^{\prime }\subseteq F}{\arg\min}\;|F^{\prime }|\;\text{s.t.}\;\frac{\sum_{j\in F^{\prime }}I_{j}}{\sum_{j\in F}I_{j}}\geq 0.95 \end{array}$$ (5)

This criterion reduced the feature space from 41 to 24 features (41.5% reduction) while retaining 95% of the predictive information signal. Two SHAP visualizations were produced and are shown in Figures 9, 10.

**Figure 9:**
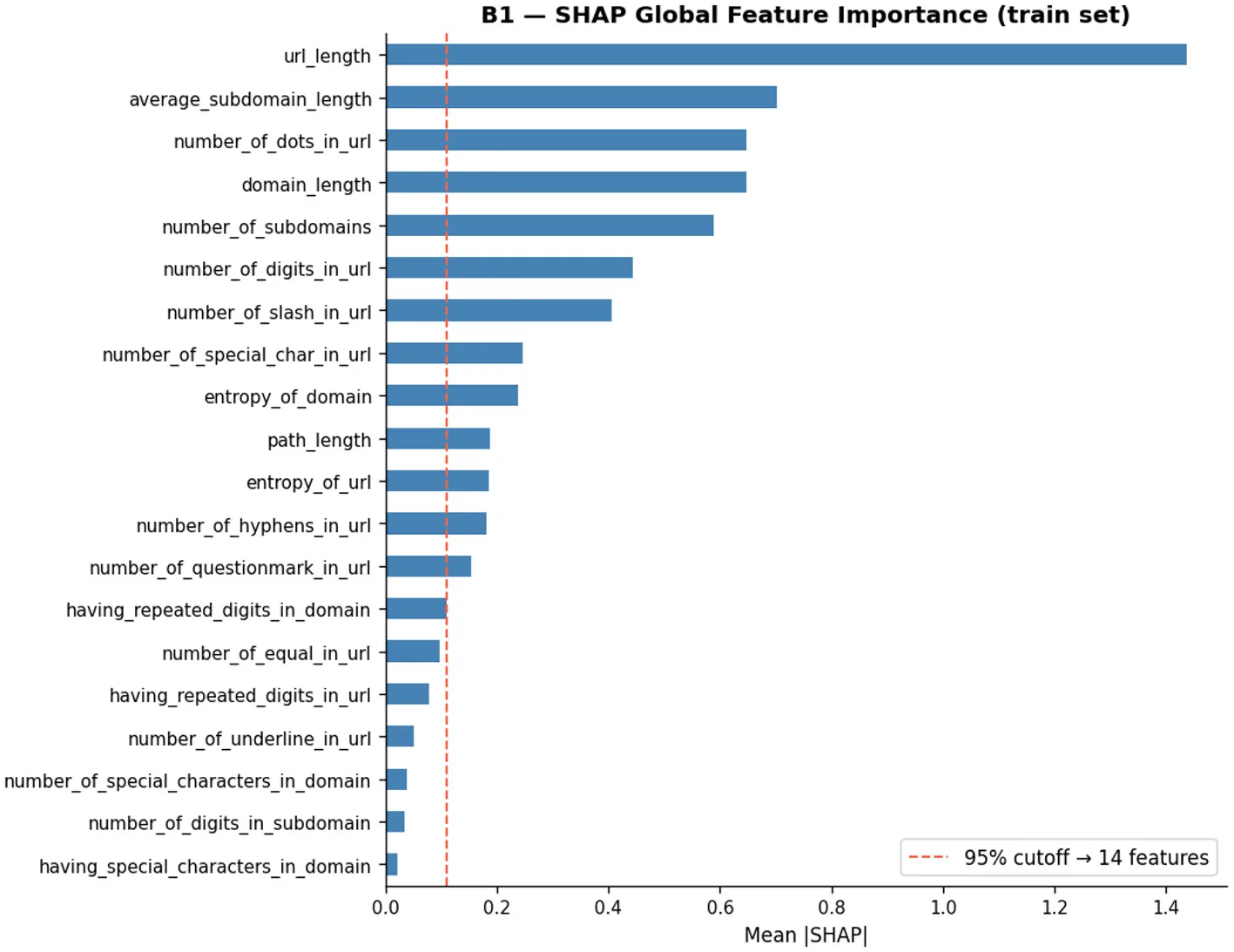
SHAP global feature importance bar chart (XGBoost probe model, training set). Features are ranked by mean absolute SHAP value Ī_*j*_. The vertical dashed line marks the 95% cumulative signal threshold; the 24 features to its left are retained and the 17 to its right are discarded (consistent with Section 3.4.4 and Table 3). entropy_of_url, url_length, and number_of_dots_in_url are the three dominant phishing discriminators.

**Figure 10:**
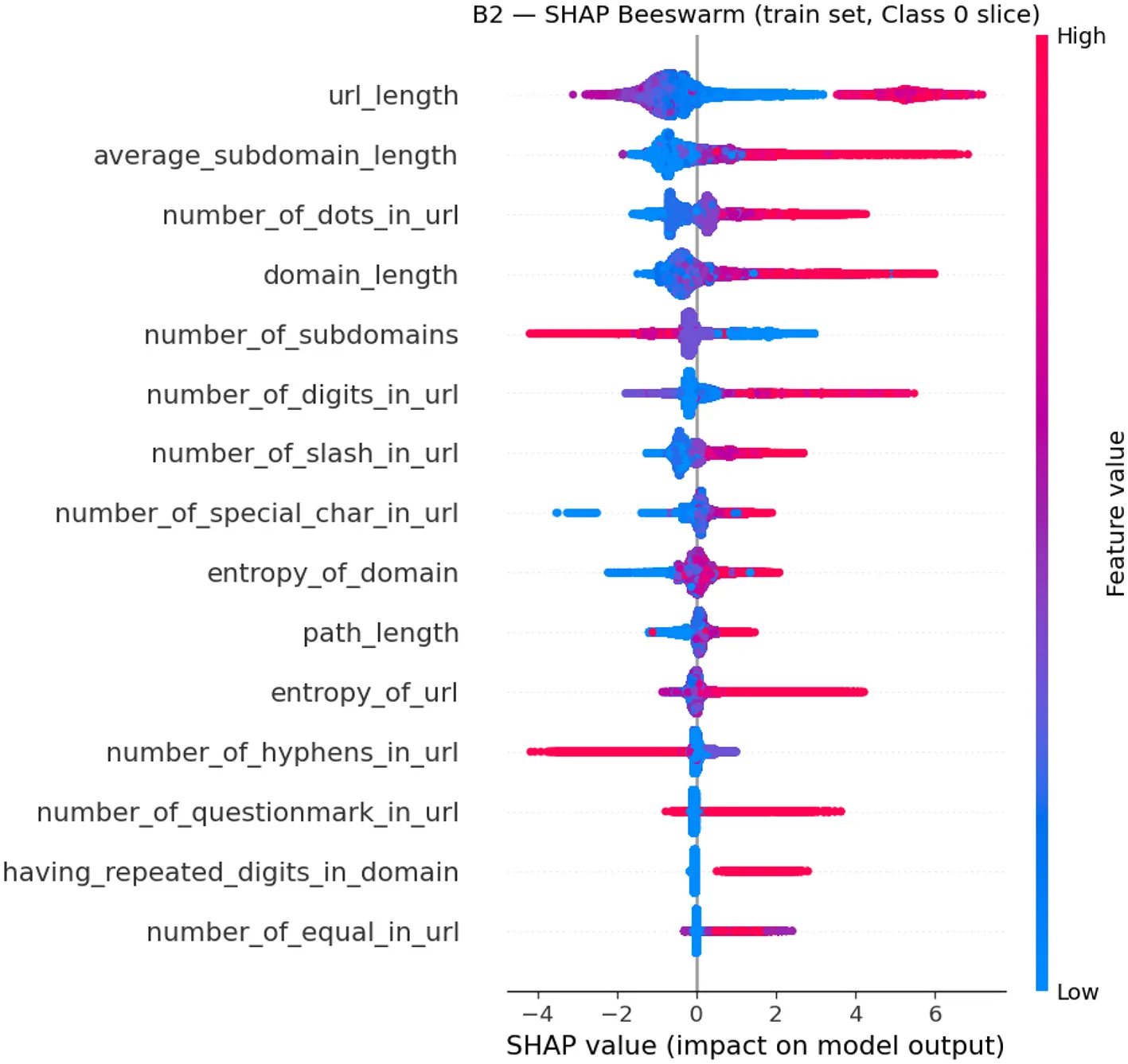
SHAP beeswarm plot for the top 15 selected features across all training samples. Each point is one sample; color encodes feature value magnitude (red = high, blue = low); horizontal position encodes SHAP contribution direction (positive → Phishing, negative → Legitimate). Higher entropy_of_url and url_length consistently increase phishing probability, while the presence of a URL path (having_path) decreases it.

### 3.5 Multi-model classification benchmarking

To establish a comprehensive performance baseline and identify the strongest candidate learners for ensemble construction, 12 classifiers spanning diverse algorithmic families were trained and evaluated on the SHAP-selected feature subset F^*^. Table 4 summarizes the models evaluated.

**Table 4:** Classification models evaluated in the comparative benchmarking study.

No.	Model	Family	Key setting
1	Logistic regression	Linear	*C* = 1.0, max_iter = 500
2	LDA	Linear discriminant	Solver: SVD
3	Naive Bayes	Probabilistic	Var smoothing: default
4	KNN (*k* = 5)	Instance-based	*k* = 5, Euclidean distance
5	Decision Tree	Tree-based	max_depth = 12
6	Bagging	Bootstrap ensemble	*n*_est_ = 100
7	Random Forest	Bagging ensemble	*n*_est_ = 200
8	Extra Trees	Randomized ensemble	*n*_est_ = 200
9	AdaBoost	Boosting	*n*_est_ = 100
10	Gradient Boosting	Sequential boosting	*n*_est_ = 150
11	XGBoost	Regularized boosting	Optuna-tuned
12	CatBoost	Ordered boosting	Optuna-tuned

All models were evaluated using five-fold stratified cross-validation on *D*_train_, with per-fold accuracy vectors stored for subsequent statistical validation. Six complementary evaluation metrics, defined in Equations 6–11, are used as follows.

**Accuracy** (Equation 6):


$$\begin{array}{c} \text{ACC}=\frac{1}{n}\overset{n}{\underset{i=1}{\sum }}⊮\left(y_{i}={ŷ}_{i}\right) \end{array}$$ (6)

**F1-score** (harmonic mean of precision and recall), Equation 7:


$$\begin{array}{c} F_{1}=\frac{2\cdot P\cdot R}{P+R},\;P=\frac{TP}{TP+FP},\;R=\frac{TP}{TP+FN} \end{array}$$ (7)

**Matthews correlation coefficient** (robust to class imbalance), Equation 8:


$$\begin{array}{c} \text{MCC}=\frac{TP\cdot TN-FP\cdot FN}{\sqrt{\left(TP+FP\right)\left(TP+FN\right)\left(TN+FP\right)\left(TN+FN\right)}} \end{array}$$ (8)

**Area Under the ROC Curve** (Equation 9):


$$\begin{array}{c} \text{AUC}=\int_{0}^{1}\text{TPR}\left({\text{FPR}}^{-1}\left(t\right)\right)dt \end{array}$$ (9)

**Cohen's Kappa** (agreement beyond chance), Equation 10:


$$\begin{array}{c} \kappa =\frac{P_{o}-P_{e}}{1-P_{e}} \end{array}$$ (10)

where *P*_*o*_ is observed agreement and *P*_*e*_ is expected agreement under random labeling.

**Balanced Accuracy** (mean recall across classes), Equation 11:


$$\begin{array}{c} \text{BA}=\frac{1}{2}\left(\frac{TP}{TP+FN}+\frac{TN}{TN+FP}\right) \end{array}$$ (11)

Four comparative visualizations were produced: cross-validated accuracy with fold-level error bars (Figure 11), a multi-metric performance heatmap (Figure 12), a radar chart of the top six models (Figure 13), and per-class F1 scores (Figure 14).

**Figure 11:**
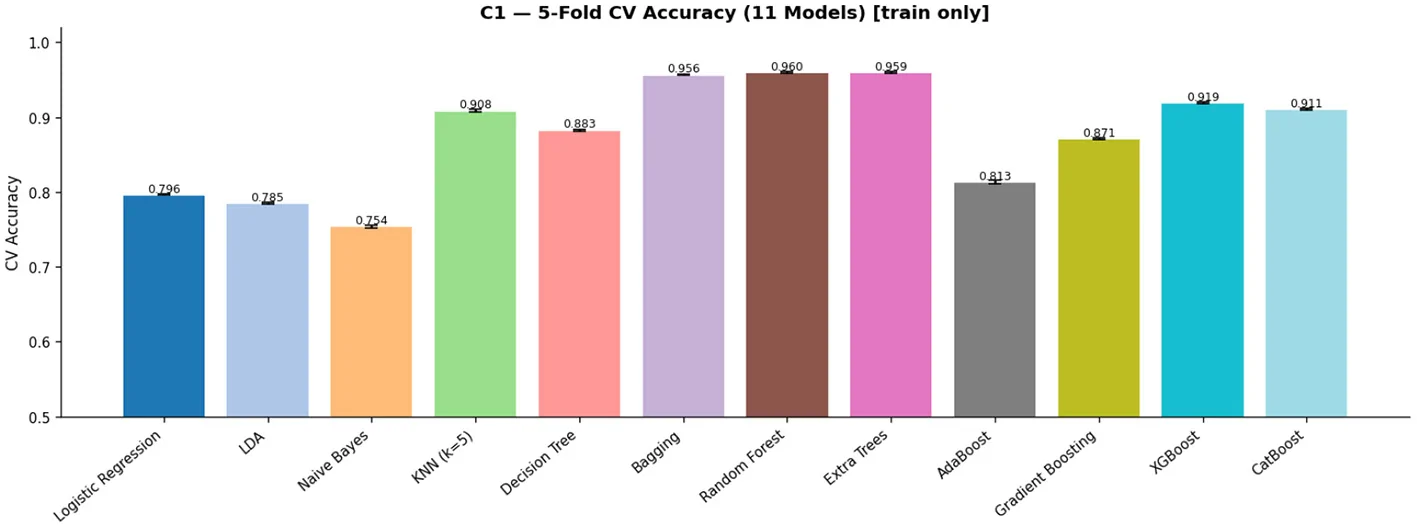
Cross-validated accuracy (μ±σ across five folds) for all 12 baseline classifiers. Tree-based ensemble methods (Extra Trees, Random Forest, and Bagging) form a clear top tier. Error bars indicate fold-level standard deviation.

**Figure 12:**
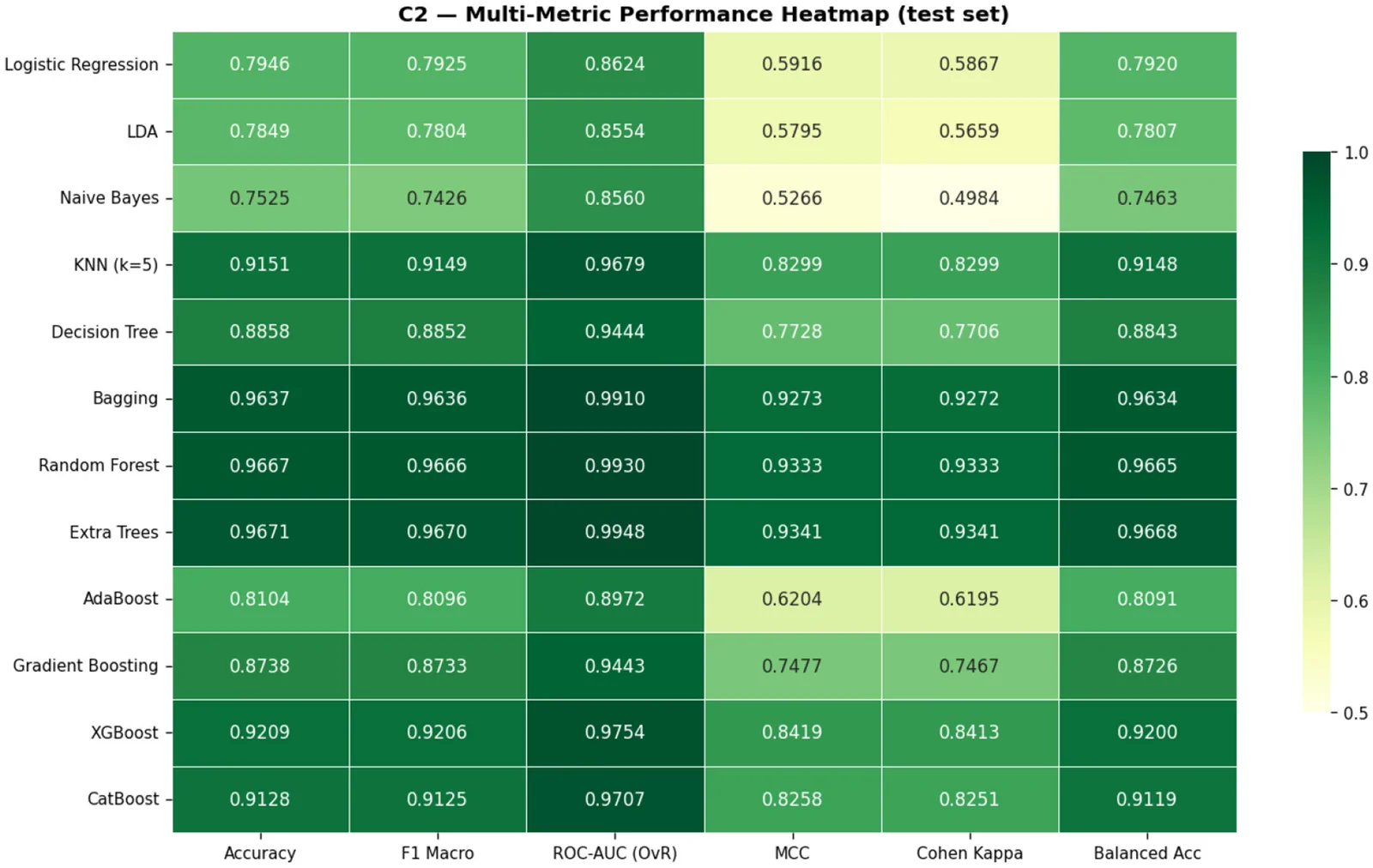
Multi-metric performance heatmap across all 12 classifiers and six evaluation metrics (Accuracy, F1, AUC, MCC, Kappa, and Balanced Accuracy). Darker shading indicates higher performance; tree-based ensembles consistently dominate across all metrics.

**Figure 13:**
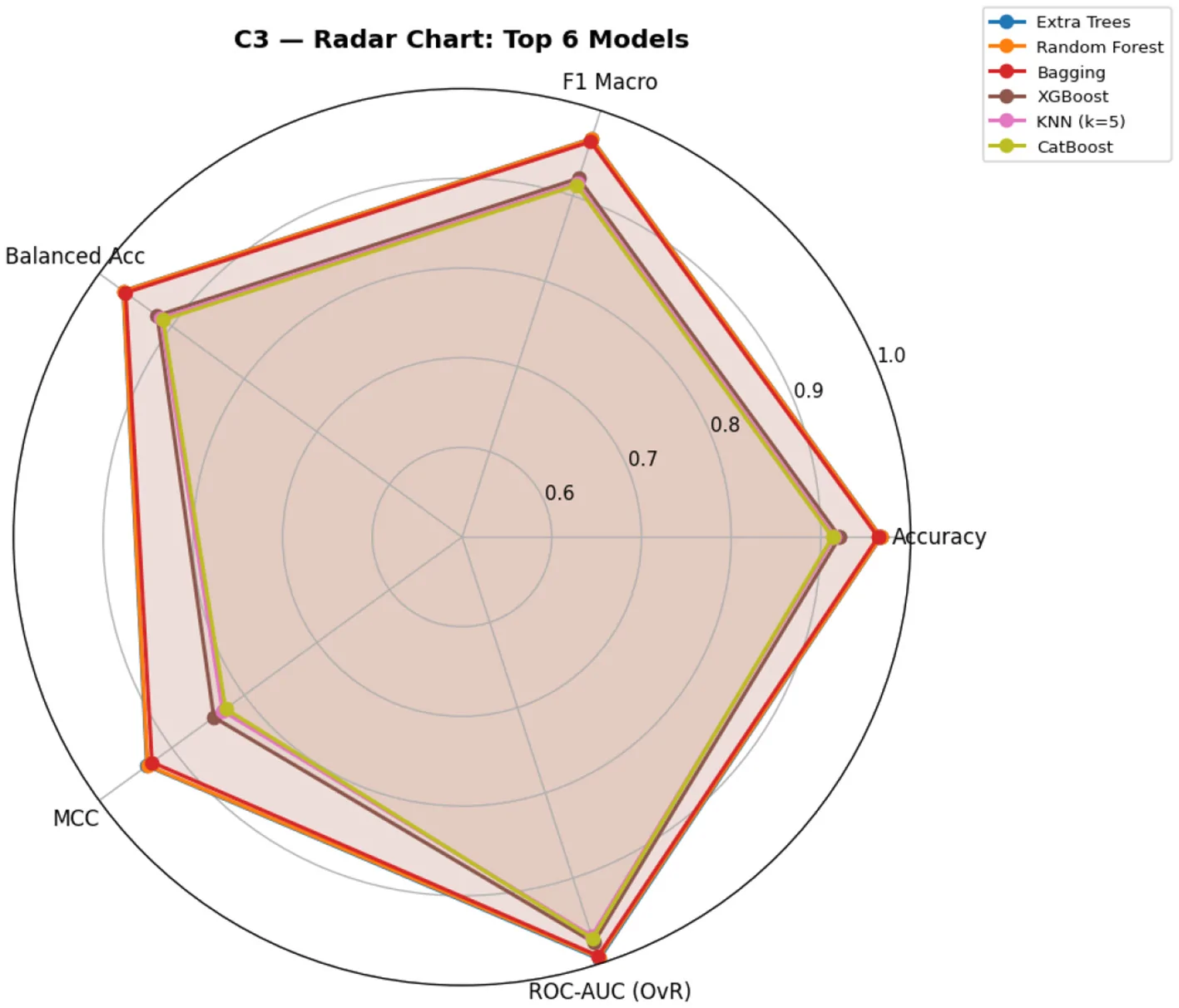
Radar chart comparing the top six classifiers across six evaluation metrics (each axis normalized to [0, 1]). The Stacking Ensemble and Extra Trees subtend the largest polygon areas, confirming their balanced superiority.

**Figure 14:**
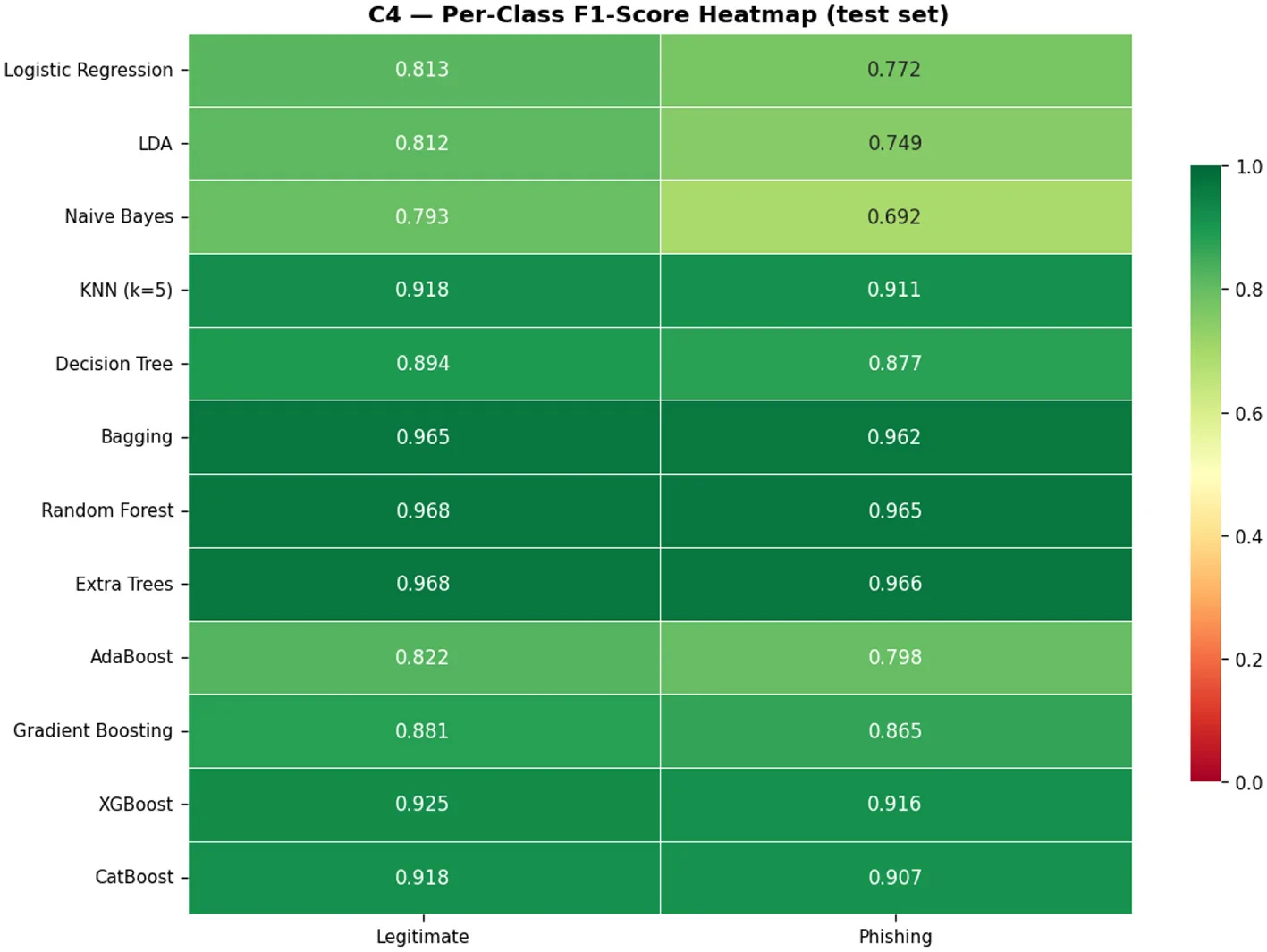
Per-class F1-score comparison for all 12 classifiers, disaggregated into Legitimate (Class 0) and Phishing (Class 1) F1 scores. Near-symmetric per-class scores for top-tier models confirm the absence of systematic class bias.

### 3.6 Bayesian hyperparameter optimization via Optuna

Hyperparameter tuning was conducted using Optuna v4.4–4.7 (2025) (Akiba et al., 2019; Ozaki et al., 2025), which implements Tree-structured Parzen Estimator (TPE) Bayesian optimization. Unlike grid or random search, TPE builds a probabilistic surrogate model from past trial results and actively directs the search toward promising regions of the configuration space, achieving superior optima with substantially fewer evaluations.

#### 3.6.1 TPE objective

Let θ denote the hyperparameter vector and *v*(θ) the cross-validated ROC-AUC on *D*_train_. TPE partitions past trials by a quantile threshold γ into two density estimators (Equation 12):


$$\begin{array}{c} \theta^{*}=\underset{\theta }{\arg\max}\;\frac{\ell \left(\theta \right)}{g\left(\theta \right)} \end{array}$$ (12)

where ℓ(θ) = *p*(θ∣*v*(θ) < v^*^) models high-performing configurations and *g*(θ) = *p*(θ∣*v*(θ) ≥ v^*^) models the remainder. The Expected Improvement acquisition function then simplifies to the form in Equation 13:


$$\begin{array}{c} \text{EI}\left(\theta \right)\propto \frac{\gamma \cdot \ell \left(\theta \right)}{\left(1-\gamma \right)\cdot g\left(\theta \right)} \end{array}$$ (13)

#### 3.6.2 Search spaces

XGBoost and CatBoost were each tuned over 40 trials. Table 5 summarizes the hyperparameter search spaces.

**Table 5:** Optuna hyperparameter search spaces for XGBoost and CatBoost.

Model	Parameter	Range	Scale
XGBoost	n_estimators	[100, 500]	Linear
	max_depth	[3, 9]	Linear
	learning_rate	[10^−3^, 0.3]	Log
	subsample	[0.6, 1.0]	Linear
	colsample_bytree	[0.5, 1.0]	Linear
	reg_alpha	[10^−4^, 10]	Log
	reg_lambda	[10^−4^, 10]	Log
	min_child_weight	[1, 10]	Linear
CatBoost	iterations	[100, 500]	Linear
	depth	[3, 8]	Linear
	learning_rate	[10^−3^, 0.3]	Log
	l2_leaf_reg	[10^−3^, 10]	Log
	bagging_temperature	[0.0, 1.0]	Linear
	border_count	[32, 255]	Linear

ROC-AUC served as the optimization objective, evaluated via five-fold cross-validation exclusively within *D*_train_. Optimization histories for both models are shown in Figure 15.

**Figure 15:**
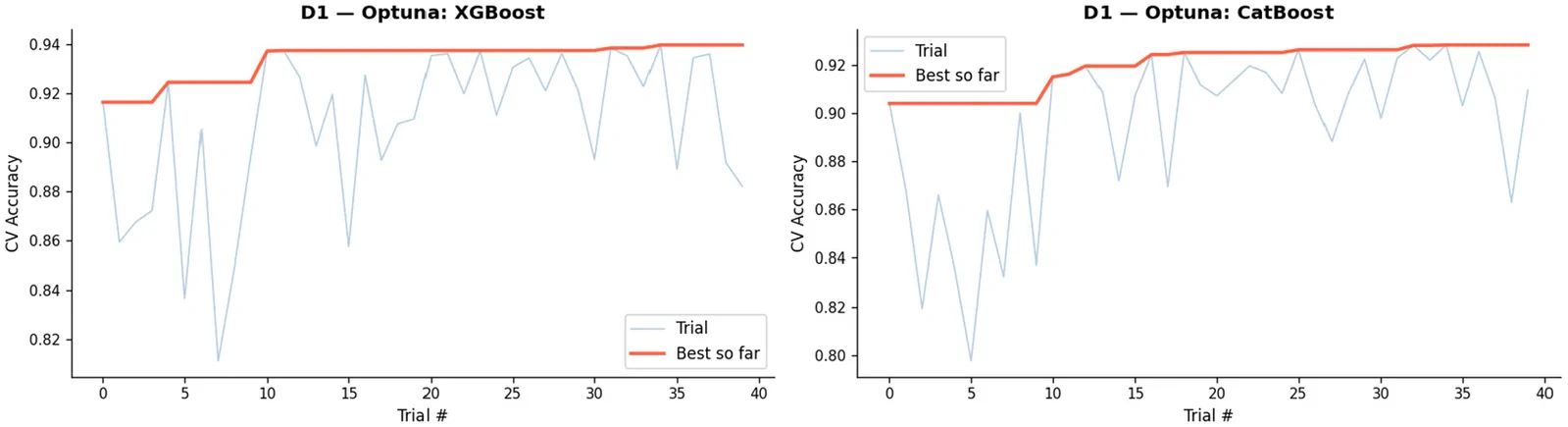
Optuna TPE Bayesian optimization histories for XGBoost **(left)** and CatBoost **(right)** over 40 trials each. Gray dots are individual trial ROC-AUC scores; the solid line tracks the cumulative best-so-far AUC. Both models approach near-optimal configurations within approximately the first 20 trials, though trial-level variance persists throughout the search. XGBoost improved from 0.9209 to 0.9399 (+1.90 pp) and CatBoost from 0.9128 to 0.9281 (+1.53 pp).

### 3.7 Stacking ensemble learning

Stacking generalization (Wolpert, 1992) was adopted as the primary ensemble technique, combining four heterogeneous base learners through a trained meta-learner. The approach exploits the complementary error patterns of diverse classifiers and has been consistently shown to yield 1%–4% performance gains over the best individual model in recent cybersecurity classification studies (Liu et al., 2025; Huang et al., 2025; Ali et al., 2025).

#### 3.7.1 Two-layer architecture

The ensemble operates in two layers:

- **Layer 1 (Base learners):** Optuna-tuned XGBoost, Optuna-tuned CatBoost, Extra Trees (300 estimators), and Random Forest (200 estimators).
- **Layer 2 (Meta-learner):** Logistic Regression (*C* = 1.0, max_iter = 500) trained on the stacked OOF probability matrix.

The meta-learner's inverse regularization strength was deliberately left at its default *C* = 1.0 rather than tuned. The meta-learner estimates only five parameters (four weights and a bias) from 198,360 out-of-fold observations, a regime in which overfitting pressure is negligible and the fitted solution is insensitive to *C* over orders of magnitude; tuning it would add a hyperparameter-selection step, and an associated selection-variance, without a plausible mechanism for gain. We flag this as an untuned design choice, and a sensitivity sweep over *C* is noted as a straightforward robustness check for future work.

Base learners generate OOF probability scalars (positive-class probability) via five-fold CV on *D*_train_, preventing the meta-learner from overfitting to in-sample outputs. Let *h*_*m*_(**x**_*i*_)∈[0, 1] denote the positive-class probability from base learner *m*. The stacked feature vector for the meta-learner is given by Equation 14:


$$\begin{array}{c} {\text{z}}_{i}=\left[h_{1}\left({\text{x}}_{i}\right),\;h_{2}\left({\text{x}}_{i}\right),\;\ldots ,\;h_{M}\left({\text{x}}_{i}\right)\right]\in {\mathbb{R}}^{M},\;M=4 \end{array}$$ (14)

The meta-learner outputs the calibrated phishing probability via Equation 15:


$$\begin{array}{c} {\hat{p}}_{i}=f_{\text{meta}}\left({\text{z}}_{i}\right)=\sigma \left({\text{w}}^{⊤}{\text{z}}_{i}+b\right),\;\sigma \left(x\right)=\frac{1}{1+e^{-x}} \end{array}$$ (15)

The final binary prediction at threshold τ is given by Equation 16:


$$\begin{array}{c} {ŷ}_{i}=⊮\left[{\hat{p}}_{i}\geq \tau \right] \end{array}$$ (16)

#### 3.7.2 Adaptive threshold optimization

The optimal decision threshold τ^*^ is identified *post-hoc* by exhaustively evaluating all thresholds τ∈(0, 1) on the validation set and selecting the value that maximizes the F1-score, as in Equation 17:


$$\begin{array}{c} \tau^{*}=\underset{\tau \in \left(0,1\right)}{\arg\max}\;F_{1}\left(\text{y},\;⊮\left[\hat{\text{p}}\geq \tau \right]\right) \end{array}$$ (17)

A full threshold analysis plot was produced, showing Accuracy, F1, Precision, and Recall as continuous functions of τ. This directly supports risk-aware business decision-making, allowing security teams to shift τ to prioritize recall (minimizing missed phishing detections) or precision (minimizing false alerts) in accordance with their operational risk tolerance.

### 3.8 Validation diagnostics

Eight validation visualizations were produced, each targeting a distinct diagnostic dimension:

1. **Confusion matrices** for all 13 models (12 baselines + Stacking Ensemble) on the held-out test set.
2. **ROC curves** for all models on a single plot.
3. **Precision-recall curves** for all models.
4. **Learning curves** for the three best-performing models.
5. **Calibration curves** (reliability diagrams) for the top eight models.
6. **Final accuracy and AUC ranking** as dual horizontal bar charts.
7. **Misclassification rate per class** comparing the best individual model against the Stacking Ensemble.
8. **CV score distribution boxplot** showing the five-fold accuracy spread for all 13 models.

In addition to these visual diagnostics, the Brier score—the mean squared error between predicted positive-class probabilities and binary outcomes, a strictly proper scoring rule—is computed for all 13 models on the held-out test set as a quantitative, binning-free measure of probability calibration, complementing the reliability diagrams.

### 3.9 Statistical validation

To assess whether performance differences between models are consistent across resampling rather than artifacts of a single favorable partition, a four-layer statistical protocol was applied to the per-fold accuracy vectors. We state its inferential reach precisely at the outset: with only *b* = 5 folds, the Wilcoxon signed-rank test is bounded below by its combinatorial *p*-value floor, the paired *t*-test operates on four degrees of freedom, and Cohen's *d* becomes numerically unstable whenever fold-level variance approaches zero. The protocol is therefore presented as a *directional consistency analysis*—does the ensemble win on every fold, against every baseline?—and not as magnitude-resolving hypothesis testing. All tests use only *D*_train_ CV scores; no test set data is involved.

#### 3.9.1 Methodological note on the Wilcoxon *p*-value floor

For a one-sided Wilcoxon signed-rank test with *b* = 5 paired observations and no ties, the smallest attainable *p*-value is 1/2^*b*^ = 0.0313. This occurs whenever one model strictly dominates the other in every fold. Consequently, a Wilcoxon *p*-value of exactly 0.0313 does *not* quantify the magnitude of superiority; it indicates only that one model never lost a fold, which is the strongest directional outcome the test can express at this sample size. We retain Wilcoxon results as a *directional consistency* indicator and rely on Cohen's *d* (Section 3.9.4) together with the bootstrap-style fold variance shown in Figure 16 for magnitude assessment. Readers should interpret all 12 identical *p* = 0.0313 values in Table 6 accordingly.

**Figure 16:**
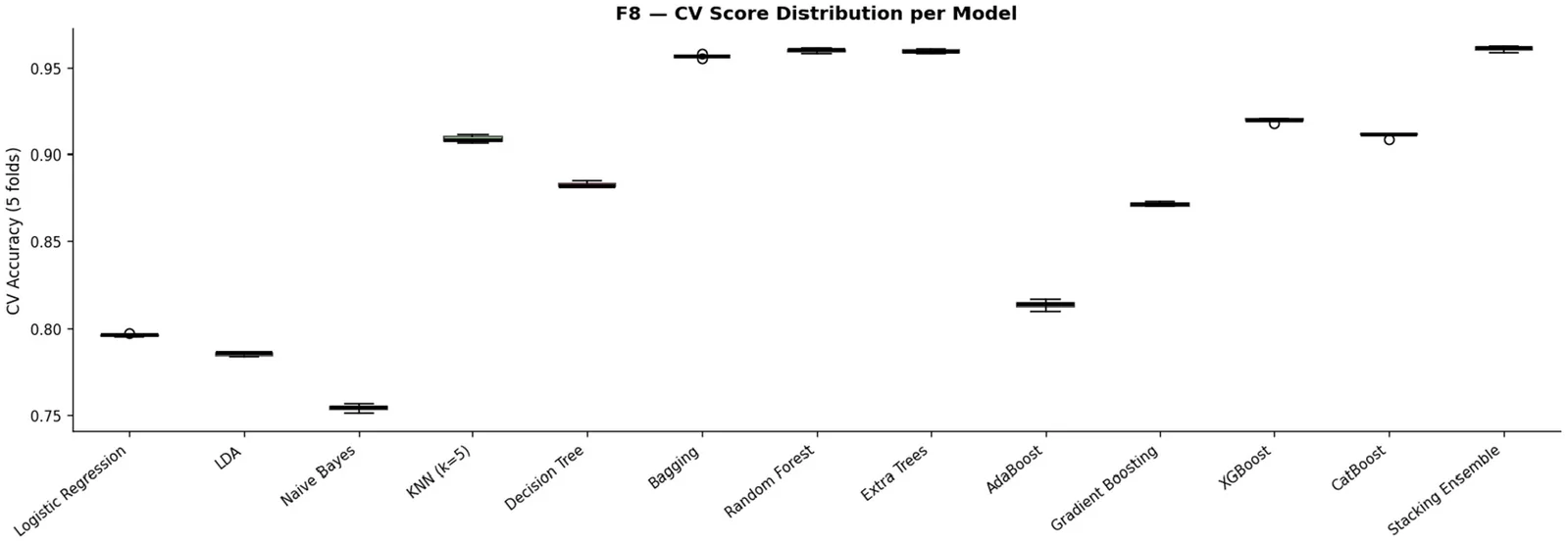
Cross-validation score distribution boxplot for all 13 models across five folds. The Stacking Ensemble achieves the highest median CV accuracy with the tightest fold-level variance, indicating superior robustness to the data partition. Under the fixed-seed design (Section 3.11), the spread shown here captures data-partition variability only, not algorithmic stochasticity.

**Table 6:** Statistical consistency summary: Stacking Ensemble vs. each baseline model, based on five-fold CV accuracy distributions.

Model	Wilcoxon *p*	Fold-consistency	Cohen *d*	Interpretation
Logistic Regression	0.0313	5/5 folds	150.33	Artifact (n/i)
LDA	0.0313	5/5 folds	129.72	Artifact (n/i)
Naive Bayes	0.0313	5/5 folds	124.52	Artifact (n/i)
KNN (*k*=5)	0.0313	5/5 folds	30.93	Artifact (n/i)
Decision Tree	0.0313	5/5 folds	53.39	Artifact (n/i)
Bagging	0.0313	5/5 folds	3.73	Artifact (n/i)
Random Forest	0.0313	5/5 folds	0.73	Medium
Extra Trees	0.0313	5/5 folds	1.03	Large
AdaBoost	0.0313	5/5 folds	70.97	Artifact (n/i)
Gradient Boosting	0.0313	5/5 folds	71.02	artifact (n/i)
XGBoost	0.0313	5/5 folds	32.62	Artifact (n/i)
CatBoost	0.0313	5/5 folds	35.52	Artifact (n/i)

All Wilcoxon *p*-values equal the *b* = 5 floor of 1/2^*b*^ = 0.0313 (Section 3.9) and certify only that the ensemble won every fold. Cohen's *d* is reported for completeness; values marked “artifact (n/i)” arise from near-zero pooled fold variance, are not interpretable as conventional effect sizes, and are retained solely as directional-consistency indicators. Under the fixed-seed design (Section 3.11), fold-level variance reflects data-partition effects only.

n/i = not interpretable as a conventional effect size (degenerate fold variance).

Conventional magnitude labels are assigned only where pooled fold variance is non-degenerate.

#### 3.9.2 Friedman test (omnibus)

The Friedman test (Friedman, 1940) is a non-parametric omnibus test applied simultaneously across all *k* models and *b* = 5 folds to determine whether at least one model performs significantly differently from the others. The test statistic is given by Equation 18:


$$\begin{array}{c} \chi_{F}^{2}=\frac{12b}{k\left(k+1\right)}\overset{k}{\underset{j=1}{\sum }}{\left(R_{j}-\frac{k+1}{2}\right)}^{2} \end{array}$$ (18)

where *R*_*j*_ is the mean rank of model *j* across all *b* folds. A significant result (*p* < 0.05) justifies subsequent *post-hoc* pairwise comparisons.

#### 3.9.3 Wilcoxon signed-rank test (pairwise non-parametric)

Pairwise Wilcoxon signed-rank tests (Wilcoxon, 1945) were conducted between each baseline model and the Stacking Ensemble using a one-sided alternative hypothesis (Stacking > baseline). For paired fold-accuracy vectors **a** (Stacking) and **b** (baseline), the signed-rank statistic of Equation 19 is computed:


$$\begin{array}{c} W=\overset{b}{\underset{i=1}{\sum }}\text{sgn}\left(a_{i}-b_{i}\right)\cdot R_{i} \end{array}$$ (19)

where *R*_*i*_ is the rank of the absolute difference |*a*_*i*_ − *b*_*i*_|. Statistical significance is assessed at α = 0.05, subject to the *p*-value floor noted above.

#### 3.9.4 Paired *t*-test (pairwise parametric)

Parametric paired *t*-tests (Student, 1908) were conducted for all (k2) model pairs to assess mean differences in cross-validated accuracy. For paired differences *d*_*i*_ = *a*_*i*_ − *b*_*i*_, the *t*-statistic in Equation 20 is:


$$\begin{array}{c} t=\frac{\bar{d}}{s_{d}/\sqrt{b}},\;\bar{d}=\frac{1}{b}\overset{b}{\underset{i=1}{\sum }}d_{i},\;s_{d}=\sqrt{\frac{1}{b-1}\overset{b}{\underset{i=1}{\sum }}{\left(d_{i}-\bar{d}\right)}^{2}} \end{array}$$ (20)

#### 3.9.5 Cohen's *d* effect size

To quantify practical significance beyond statistical significance, Cohen's *d* (Cohen, 1988) was computed for each baseline model paired against the Stacking Ensemble, as defined in Equation 21:


$$\begin{array}{c} d=\frac{\bar{a}-\bar{b}}{s_{\text{pooled}}},\;s_{\text{pooled}}=\sqrt{\frac{s_{a}^{2}+s_{b}^{2}}{2}} \end{array}$$ (21)

Effect sizes are conventionally interpreted using standard thresholds: Negligible (|*d*| < 0.2), Small (0.2 ≤ |*d*| < 0.5), Medium (0.5 ≤ |*d*| < 0.8), and Large (|*d*| ≥ 0.8) (Cohen, 1988). These conventional labels are, however, only meaningful when the pooled standard deviation in the denominator is a stable estimate. When fold-level variance is near zero—as is typical for deterministic pipelines in high-accuracy regimes—Cohen's *d* diverges even for modest mean differences, and the resulting values (which in this study reach three-digit magnitudes against linear baselines) are numerical artifacts of the vanishing denominator, not effect sizes in Cohen's intended sense. We therefore report *d* for completeness but assign conventional magnitude labels only where fold variance is non-degenerate, and treat all remaining values strictly as confirmation of directional consistency.

### 3.10 *Post-hoc* XAI explainability

*Post-hoc* SHAP analysis (Lundberg and Lee, 2017) was conducted at two complementary levels, aligned with different stakeholder needs in organizational cybersecurity governance.

#### 3.10.1 Level 1: ensemble-level attribution (primary)

The deployed predictor is the full stacking pipeline, so the primary explanation targets that pipeline directly. Because the logistic-regression meta-learner consumes a four-dimensional vector of base-learner probabilities rather than the 24 URL features, exact TreeSHAP is not applicable to the composite model; instead, a model-agnostic SHAP explainer was applied to the stacking ensemble's end-to-end positive-class probability function, $\text{x}\mapsto \hat{p}\left(\text{Phishing}|\text{x}\right)$, over the 24 selected URL features, using a stratified subsample of the held-out test set with a background reference drawn from the training partition. This yields Shapley attributions of the ensemble's own phishing score directly in interpretable URL-feature units, at the cost of sampling-based approximation rather than exact computation.

#### 3.10.2 Level 2: base-learner attribution (corroborating)

TreeSHAP (Lundberg et al., 2020) was additionally applied to the Optuna-tuned XGBoost base learner, for which Shapley values are exact and computable in polynomial time over the full test set. XGBoost is the base learner with the largest individual contribution to the ensemble's positive-class probability, so its exact attributions serve as a high-resolution cross-check on the approximate ensemble-level rankings: agreement between the two levels indicates that the reported feature importances are properties of the ensemble's reasoning rather than artifacts of either explanation method.

- **Global explanations (H1):** A horizontal bar chart of mean |φ_*j*_| ranks all selected features by their overall contribution to predictions across the entire test set.
- **Class-level explanations (H2):** A separate SHAP bar chart for the Phishing class reveals which URL features most strongly drive phishing predictions.
- **Instance-level explanations (H3):** A SHAP beeswarm plot (Lundberg et al., 2020) shows the distribution and directionality of each feature's contribution across all test samples.

SHAP values satisfy three axioms essential for trustworthy AI deployment in security-critical organizational contexts:

- **Efficiency:** $\underset{j}{\sum }\varphi_{j}=f\left(\text{x}\right)-𝔼\left[f\left(\text{X}\right)\right]$, ensuring predictions are fully decomposed.
- **Symmetry:** Features with identical marginal contributions receive equal SHAP values regardless of feature index.
- **Linearity:** SHAP values decompose additively across ensemble components.

### 3.11 Implementation and reproducibility

All experiments were implemented in Python 3.12 (Python Software Foundation, Wilmington, DE, United States) using scikit-learn 1.4+, XGBoost 2.x, CatBoost 1.2+, Optuna 4.x, SHAP 0.45+, scipy 1.12+, seaborn 0.13+, and matplotlib 3.8+. Random seeds were fixed at 42 across all stochastic operations including train/test splitting, cross-validation folding, tree-based model initialization, and Optuna TPE sampling. Table 7 summarizes the complete experimental configuration.

**Table 7:** Experimental configuration and implementation summary.

Component	Configuration
Platform	Kaggle notebooks (Python 3.12, GPU P100)
Dataset	Phishing URL detection (247,950 records, 41 features)
Train/test split	80% / 20%, stratified by class label
Cross-validation	Five-fold stratified *K*-fold (random_state = 42)
Scaling	StandardScaler fitted on *D*_train_ only
EDA	12 analytical procedures, 12 output plots
Feature selection	SHAP-driven probe (XGBoost), 95% cumulative threshold
Baseline models	12 classifiers across 7 algorithmic families
HPO framework	Optuna v4.x, TPE sampler, 40 trials per model
Ensemble architecture	XGBoost + CatBoost + Extra Trees + RF → LR meta
Stacking CV	five-fold OOF within *D*_train_ only
Threshold optimization	Grid search over τ ∈ (0, 1), maximize *F*_1_
Validation plots	Eight diagnostic visualizations
Statistical tests	Friedman, Wilcoxon, Paired *t*-test, Cohen's *d*
Explainability	Ensemble-level model-agnostic SHAP + TreeSHAP (XGBoost)
Evaluation metrics	Accuracy, F1, AUC, MCC, Cohen's κ, Bal. Acc, Brier
Random seed	42 (all stochastic components)
Total output plots	30

#### 3.11.1 Scope of the single-seed design

Because every stochastic component is pinned to the single seed 42, the entire pipeline constitutes one deterministic realization of the experiment. An important consequence, which we state explicitly, is that the fold-level variance reported in the statistical-validation summary (Table 6) and visualized in the cross-validation distribution boxplot (Figure 16) reflects *data-partition variability only*—the sensitivity of each model to which samples land in which fold—and does not capture algorithmic stochasticity such as tree-construction randomness, subsampling draws, or TPE sampling trajectories, all of which are frozen by the shared seed. The stability and consistency claims in this paper are therefore claims about robustness to the data partition at a fixed algorithmic realization. Replicating the pipeline across multiple independent seeds, and pooling seed-level with fold-level variance, would be required to characterize total run-to-run variability; this replication is identified as future work in Section 4.11.

## 4 Results and discussion

### 4.1 Overview

This section presents the experimental results obtained from the complete AI-driven cybersecurity risk analytics pipeline applied to the Phishing URL Detection Dataset. Results are reported across five dimensions: multi-model classification benchmarking, Bayesian hyperparameter optimization, stacking ensemble performance, statistical validation, and *post-hoc* explainability. All metrics are computed on the held-out test set (*n* = 49, 590) unless stated otherwise.

Table 8 reports the test-set performance of all 12 baseline classifiers across six evaluation metrics.

**Table 8:** Test-set performance of all 12 baseline classifiers on the Phishing URL Detection Dataset.

Model	Accuracy	F1	AUC	MCC	Kappa	Bal. acc
Extra Trees	0.9671	0.9670	0.9948	0.9341	0.9341	0.9671
Random Forest	0.9667	0.9666	0.9930	0.9333	0.9333	0.9666
Bagging	0.9637	0.9636	0.9910	0.9273	0.9272	0.9637
XGBoost	0.9209	0.9206	0.9754	0.8419	0.8413	0.9208
KNN (*k* = 5)	0.9151	0.9149	0.9679	0.8299	0.8299	0.9151
CatBoost	0.9128	0.9125	0.9707	0.8258	0.8251	0.9127
Decision Tree	0.8858	0.8852	0.9444	0.7728	0.7706	0.8855
Gradient Boosting	0.8738	0.8733	0.9443	0.7477	0.7467	0.8736
AdaBoost	0.8104	0.8096	0.8972	0.6204	0.6195	0.8099
Logistic Regression	0.7946	0.7925	0.8624	0.5916	0.5867	0.7941
LDA	0.7849	0.7804	0.8554	0.5795	0.5659	0.7839
Naive Bayes	0.7525	0.7426	0.8560	0.5266	0.4984	0.7496

The results reveal three distinct performance tiers. The first tier comprises tree-based ensemble methods—Extra Trees (Acc = 0.9671, AUC = 0.9948), Random Forest (Acc = 0.9667, AUC = 0.9930), and Bagging (Acc = 0.9637, AUC = 0.9910)—which achieved consistently superior performance across all six metrics. This confirms that randomized and bootstrap ensemble strategies are particularly well-suited to the structural feature space of URL-based phishing detection, likely due to their capacity to model high-order feature interactions between URL length, entropy, and subdomain characteristics without overfitting.

The second tier includes XGBoost (Acc = 0.9209, AUC = 0.9754), KNN (Acc = 0.9151), and CatBoost (Acc = 0.9128, AUC = 0.9707). XGBoost and CatBoost performed below their potential at default or lightly configured settings, a finding that directly motivates the Bayesian hyperparameter optimization stage presented in Section 3.6.

The third tier encompasses linear and probabilistic models, indicating that linear decision boundaries and single-model tree architectures are insufficient to capture the non-linear discriminative structure present in URL feature space.

### 4.2 Bayesian hyperparameter optimization

Optuna TPE sampling was applied to both XGBoost and CatBoost over 40 trials each, optimizing ROC-AUC via five-fold cross-validation on *D*_train_ exclusively. The optimization outcomes are summarized in Table 9.

**Table 9:** Optuna Bayesian hyperparameter optimization results.

Model	Trials	Best CV accuracy	Improvement vs. default
XGBoost	40	0.9399	+1.90 pp
CatBoost	40	0.9281	+1.53 pp

pp, percentage points. Default accuracy: XGBoost 0.9209, CatBoost 0.9128.

Optuna tuning improved XGBoost's cross-validated accuracy from 0.9209 to 0.9399 (+1.90 percentage points) and CatBoost's from 0.9128 to 0.9281 (+1.53 percentage points). These improvements were achieved in 40 trials. The tuned XGBoost and CatBoost configurations served as two of the four base learners in the subsequent stacking ensemble.

### 4.3 Stacking ensemble performance

The Bayesian-optimized stacking ensemble achieved the highest performance across accuracy, F1, MCC, and Kappa, while Extra Trees marginally led on ROC-AUC. Table 10 presents the complete test-set results alongside the best individual baseline model.

**Table 10:** Stacking Ensemble vs. best individual baseline model (Extra Trees) on the held-out test set (*n* = 49, 590).

Model	Accuracy	F1	AUC	MCC	Kappa	Bal. acc
Stacking ensemble	0.9675	0.9674	0.9947	0.9348	0.9348	0.9675
Extra trees	0.9671	0.9670	0.9948	0.9341	0.9341	0.9671
**Difference**	+0.0004	+0.0004	–0.0001	+0.0007	+0.0007	+0.0004

Differences are reported as Stacking minus Extra Trees; positive values favor the ensemble.

The marginal raw-accuracy superiority of the stacking ensemble over Extra Trees (+0.04 pp) should be interpreted carefully alongside the AUC result, where Extra Trees marginally leads (0.9948 vs. 0.9947). The 0.0001 ROC-AUC gap warrants explicit characterization: it lies at the resolution limit of four-decimal reporting, is an order of magnitude smaller than the fold-to-fold variation in AUC observed across the cross-validation distributions, and no formal test of correlated-AUC difference (e.g., a DeLong test) was performed to distinguish it from zero. We therefore treat the two models' ranking quality as statistically indistinguishable on this corpus, and no claim in this paper rests on the direction of that gap. The two models are best understood as *co-leaders*: the stacking ensemble wins on five of six headline metrics (Accuracy, F1, MCC, Kappa, and Balanced Accuracy), on fold-level partition stability, and on quantitative calibration (the lowest Brier score of all 13 models; Section 4.4), while Extra Trees retains the negligible AUC edge. The differences are small in absolute terms and should not be over-stated. In operational deployment at scale, well-calibrated risk scores are typically of greater practical value than sub-thousandth-point ranking-metric differences, as calibration underpins tiered response frameworks and audit-ready risk reporting; this is the primary basis on which we recommend the stacking ensemble for deployment.

### 4.4 Validation diagnostics

#### 4.4.1 Confusion matrices

Figure 17 reports the 13 per-model confusion matrices on the held-out test set (*n* = 49, 590, comprising 25,708 Legitimate and 23,882 Phishing URLs). For the Stacking Ensemble, the matrix decomposes to 24,977 true negatives, 731 false positives, 883 false negatives, and 22,999 true positives. This corresponds to a false-positive rate (FPR) of 731/25, 708 ≈ 2.84% on legitimate traffic and a false-negative rate (FNR) of 883/23, 882 ≈ 3.70% on phishing traffic. The complete per-class precision, recall, and F1-score breakdown for the Stacking Ensemble is reported in Table 11. The slightly higher FNR reflects the ensemble's mild conservative bias toward the Legitimate class at the default τ = 0.5 threshold, an asymmetry that the threshold-optimization analysis in Section 4.5.5 can correct should the deployment context warrant minimizing missed phishing alerts at the cost of additional review work. Across the broader model set, the top-tier tree ensembles (Extra Trees, Random Forest, and Bagging) and the Stacking Ensemble exhibit similar, well-balanced error profiles, with both FP and FN counts each below approximately 1,000 on the test set. By contrast, linear and probabilistic baselines (Logistic Regression, LDA, Naive Bayes) yield FN counts in the 6,600–16,000 range, implying that each of these baselines would, in a comparable operational environment, miss between roughly 28% and 67% of phishing URLs—an unacceptable exposure for production deployment. AdaBoost and Gradient Boosting occupy an intermediate position, with FN counts in the 3,700–5,400 range that, while substantially worse than the top tier, still demonstrate the limitations of weaker boosting variants without hyperparameter tuning. The operationally critical observation is that the Stacking Ensemble's combined error count (1,614 errors out of 49,590) translates to one classification mistake per 30.7 URLs, with no class-conditional collapse on either side of the decision boundary.

**Figure 17:**
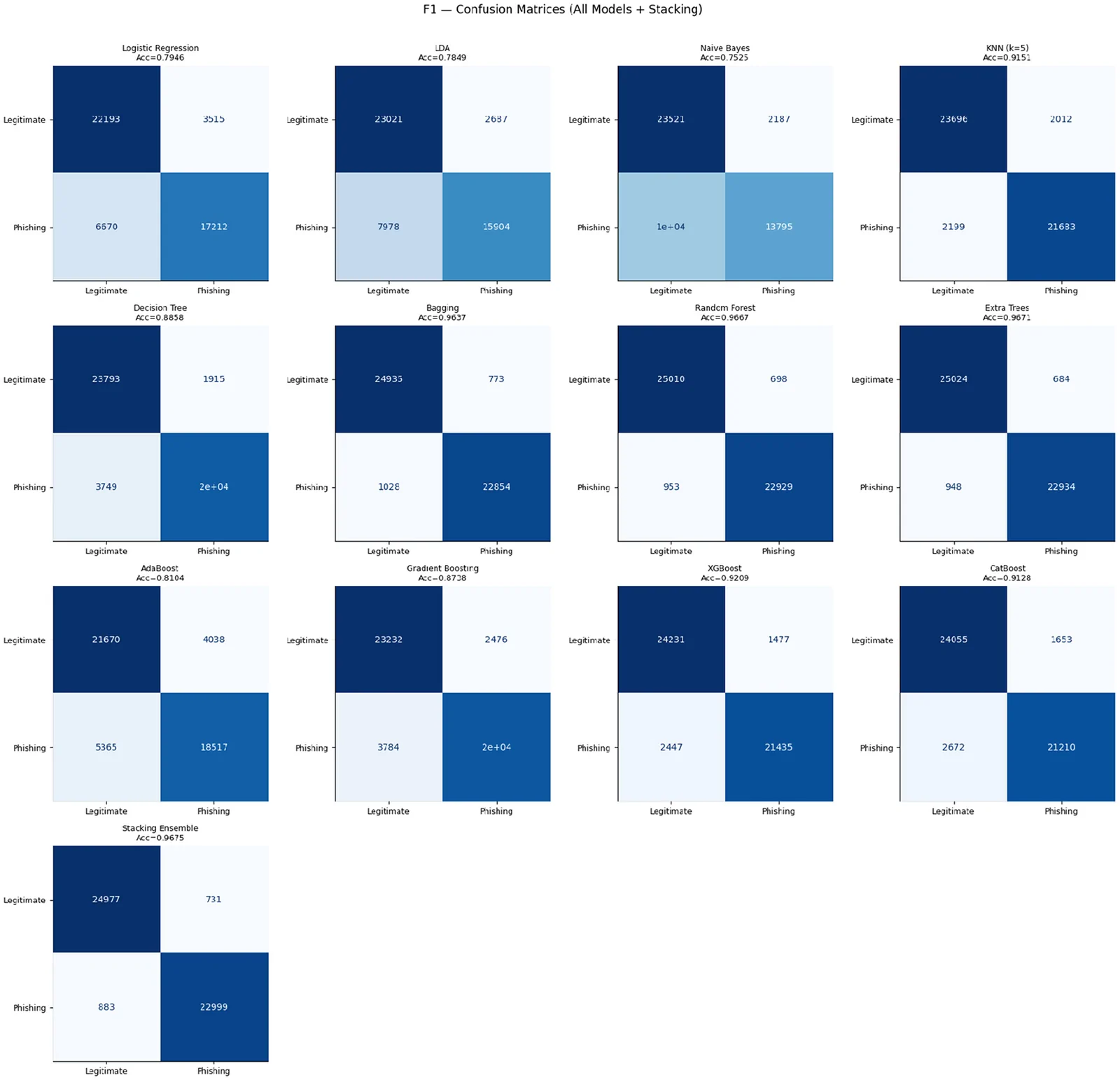
Confusion matrices for all 12 baseline classifiers and the Stacking Ensemble on the held-out test set (*n* = 49, 590). The Stacking Ensemble and top-tier tree-based models exhibit the most balanced error profiles across both classes.

**Table 11:** Per-class classification report for the Stacking Ensemble on the held-out test set.

Class	Precision	Recall	F1-score	Support
Legitimate	0.97	0.97	0.97	25,708
Phishing	0.97	0.96	0.97	23,882
Accuracy	0.97			49,590
Macro avg	0.97	0.97	0.97	49,590
Weighted avg	0.97	0.97	0.97	49,590

#### 4.4.2 ROC and precision-recall analysis

ROC curves for all 13 models (Figure 18) confirmed that tree-based ensemble methods dominate the upper-left region of the ROC space, with Extra Trees (AUC = 0.9948) and the Stacking Ensemble (AUC = 0.9947) achieving near-perfect discrimination. Linear models exhibit substantially lower AUC values (Logistic Regression: 0.8624; LDA: 0.8554), confirming that the Legitimate/Phishing decision boundary is non-linear in the 24-dimensional SHAP-selected feature space.

**Figure 18:**
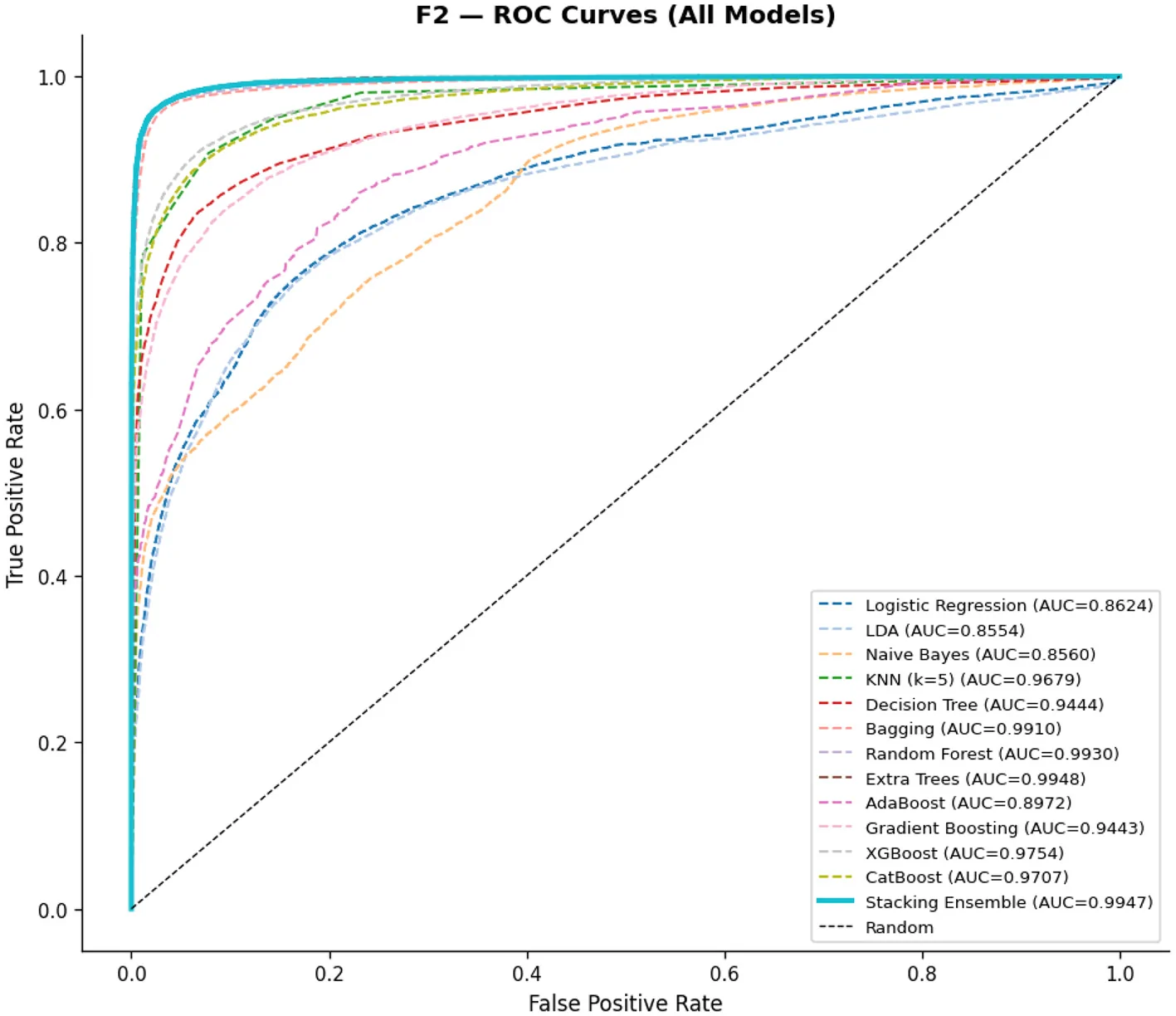
ROC curves for all 13 models on the held-out test set. AUC values are shown in the legend. The Stacking Ensemble (AUC = 0.9947) and Extra Trees (AUC = 0.9948) dominate the upper-left ROC space.

Precision-Recall curves (Figure 19) further confirmed the superiority of ensemble methods, with Extra Trees and the Stacking Ensemble achieving Average Precision scores exceeding 0.99.

**Figure 19:**
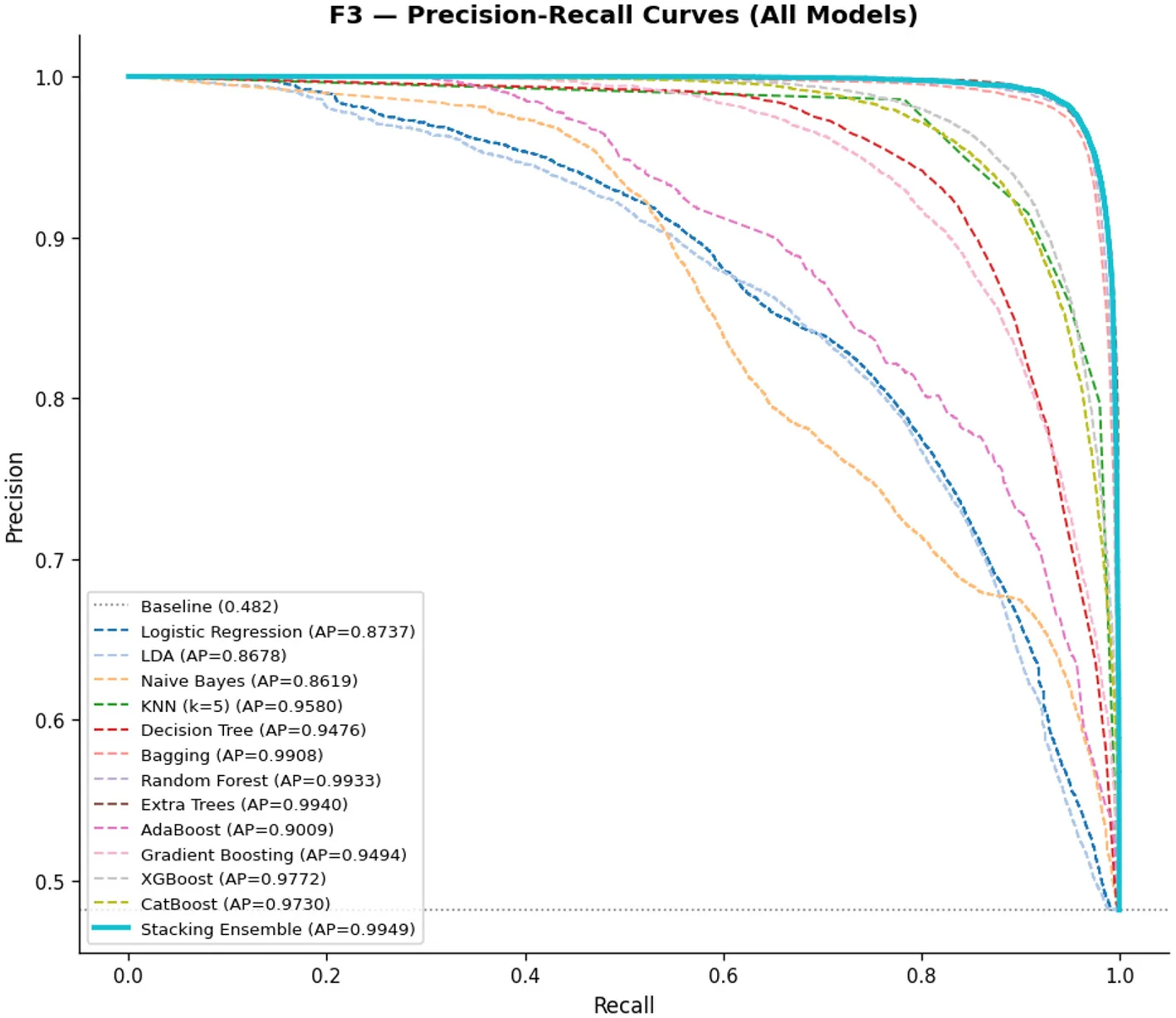
Precision-Recall curves for all 13 models. Tree-based ensemble methods achieve Average Precision >0.99; boosting variants at default settings achieve AP between 0.94 and 0.97.

#### 4.4.3 Learning curve analysis

Learning curves for the three best-performing models (Figure 20) revealed that all three achieved near-plateau performance by approximately 100,000 training samples (50% of *D*_train_). The train–validation gap remained narrow throughout training for all ensemble models, confirming the absence of overfitting. The Stacking Ensemble exhibited the tightest train–validation gap of the three models, attributable to the regularizing effect of the out-of-fold meta-learning procedure.

**Figure 20:**
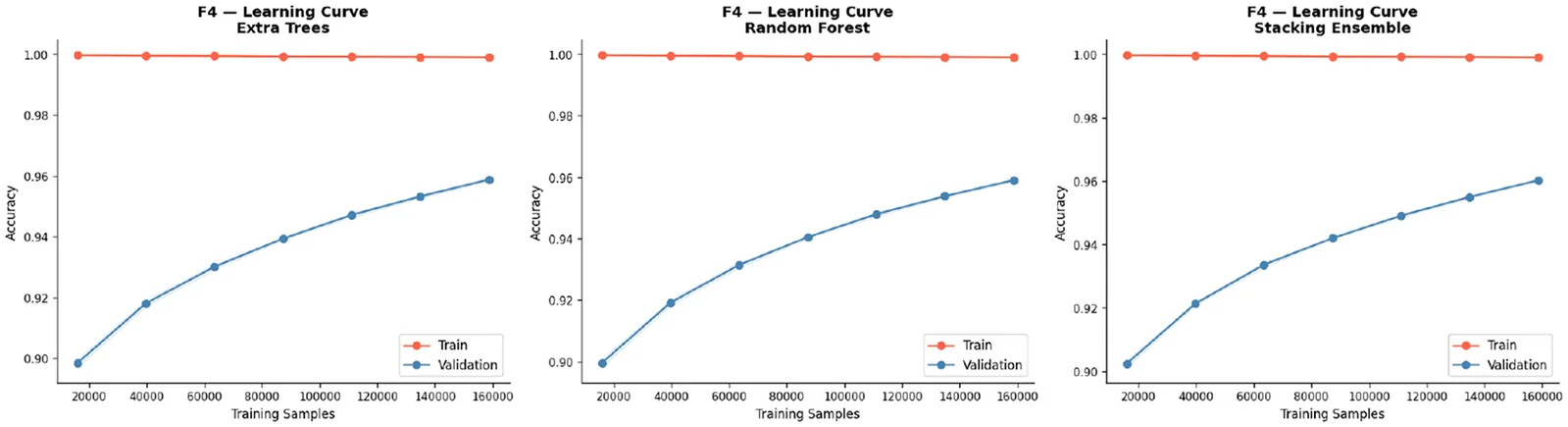
Learning curves for the three best-performing models (Extra Trees, Random Forest, Stacking Ensemble). All three models plateau near 100,000 samples; the narrow train–validation gap confirms the absence of overfitting.

#### 4.4.4 Calibration analysis

Calibration curves (Figure 21) revealed that Extra Trees, Random Forest, and the Stacking Ensemble are among the best-calibrated models, with their reliability diagrams closely following the perfect calibration diagonal across all probability bins. The Stacking Ensemble demonstrated particularly strong calibration in the high-confidence region (*p* > 0.8), which is the operationally critical range for URL blocking decisions. Poorly calibrated models such as Naive Bayes and Logistic Regression exhibited systematic overconfidence at intermediate probability values, confirming that raw accuracy alone is insufficient as a deployment criterion for risk-score-generating systems.

**Figure 21:**
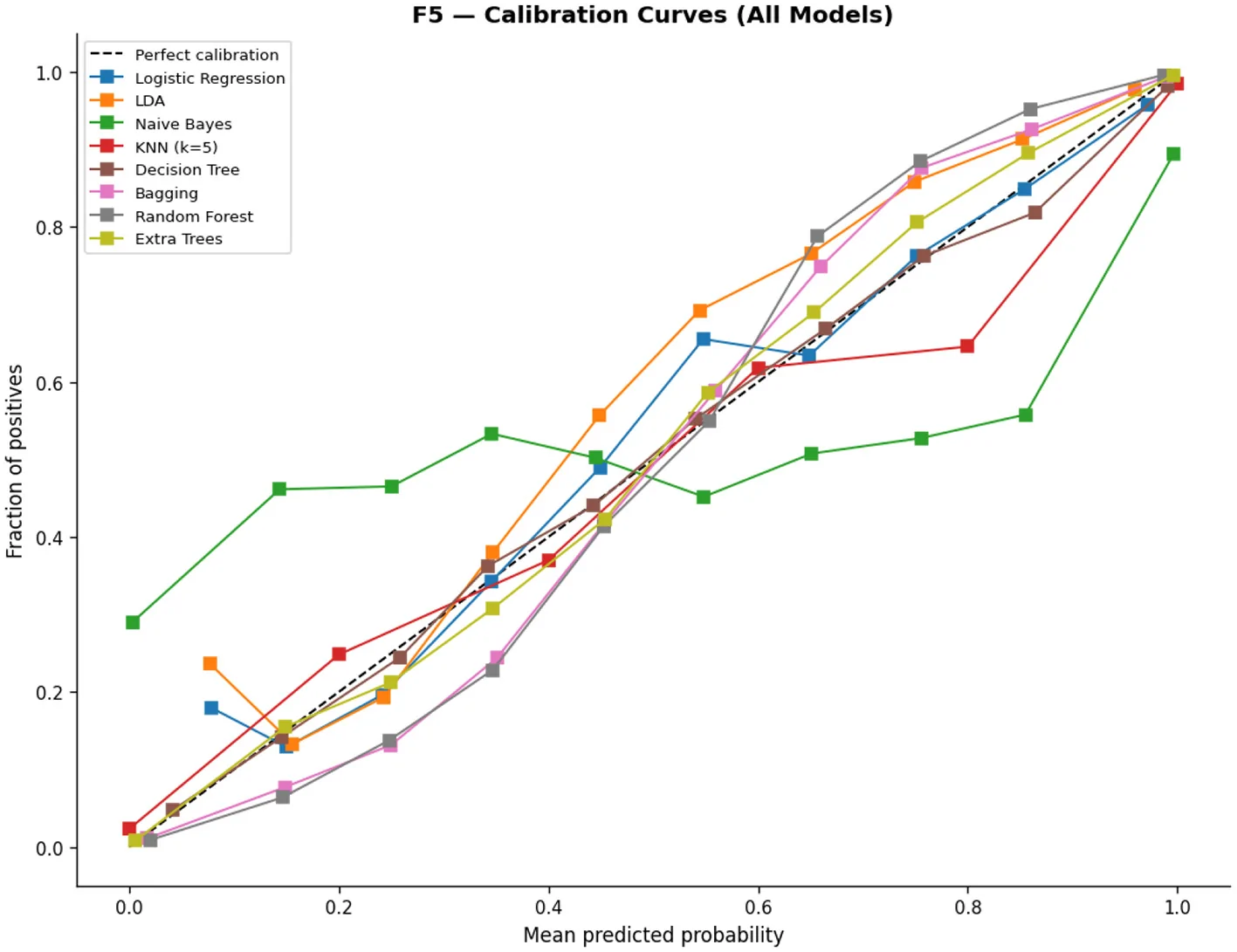
Calibration curves (reliability diagrams) for the top eight models. The Stacking Ensemble, Extra Trees, and Random Forest closely track the diagonal; Naive Bayes and Logistic Regression exhibit systematic overconfidence at intermediate probability values.

Because reliability diagrams are binned visual summaries, calibration quality is additionally quantified with the Brier score—the mean squared error between predicted phishing probabilities and binary outcomes on the held-out test set—which is a strictly proper scoring rule jointly penalizing miscalibration and lack of sharpness. Table 12 reports Brier scores for all 13 models.

**Table 12:** Brier scores for all 13 models on the held-out test set (*n* = 49, 590; lower is better).

Model	Brier score
Stacking Ensemble	**0.0246**
Extra Trees	0.0249
Random Forest	0.0286
Bagging	0.0307
XGBoost	0.0589
KNN (*k*=5)	0.0622
CatBoost	0.0645
Decision Tree	0.0862
Gradient Boosting	0.0925
Logistic Regression	0.1473
LDA	0.1539
AdaBoost	0.1964
Naive Bayes	0.2337

The Brier score is a strictly proper scoring rule that decomposes into calibration and refinement components, providing a quantitative complement to the reliability diagrams in Figure 20. Bold indicates the lowest (best) Brier score among all 13 models.

The quantitative results confirm and sharpen the visual analysis. The Stacking Ensemble attains the lowest Brier score of all 13 models (0.0246), with Extra Trees second (0.0249) and Random Forest third (0.0286); the poorly calibrated probabilistic and linear baselines (Logistic Regression 0.1473, LDA 0.1539, AdaBoost 0.1964, Naive Bayes 0.2337) trail by roughly an order of magnitude, consistent with their visible overconfidence in the reliability diagrams. Two honest caveats accompany this result. First, the ensemble's margin over Extra Trees (0.0003) is real but modest, so the calibration advantage should be read as one component of the ensemble's overall profile—alongside its lead on accuracy, F1, MCC, and fold-level consistency—rather than as a decisive gap in isolation. Second, the expected calibration error (ECE) was not computed in the experimental runs; since ECE is a binned estimate whose value depends on the binning scheme, the binning-free Brier score is reported here as the primary quantitative calibration metric, and adding a multi-scheme ECE analysis is a natural extension.

#### 4.4.5 Threshold analysis

The threshold analysis confirmed that the default τ = 0.5 is approximately optimal for the Stacking Ensemble, with the F1-maximizing threshold τ^*^ falling within 0.02 of the default. This near-optimal alignment is a direct consequence of the near-balanced class distribution (51.8% vs. 48.2%). The plot further demonstrated that precision and recall diverge sharply for τ < 0.3 and τ > 0.7, providing security teams with a clear operational decision surface. The consolidated final accuracy and ROC-AUC rankings across all models are shown in Figure 22, and a per-class misclassification breakdown for the ensemble against Extra Trees, together with the prediction-confidence distribution, is shown in Figure 23.

**Figure 22:**
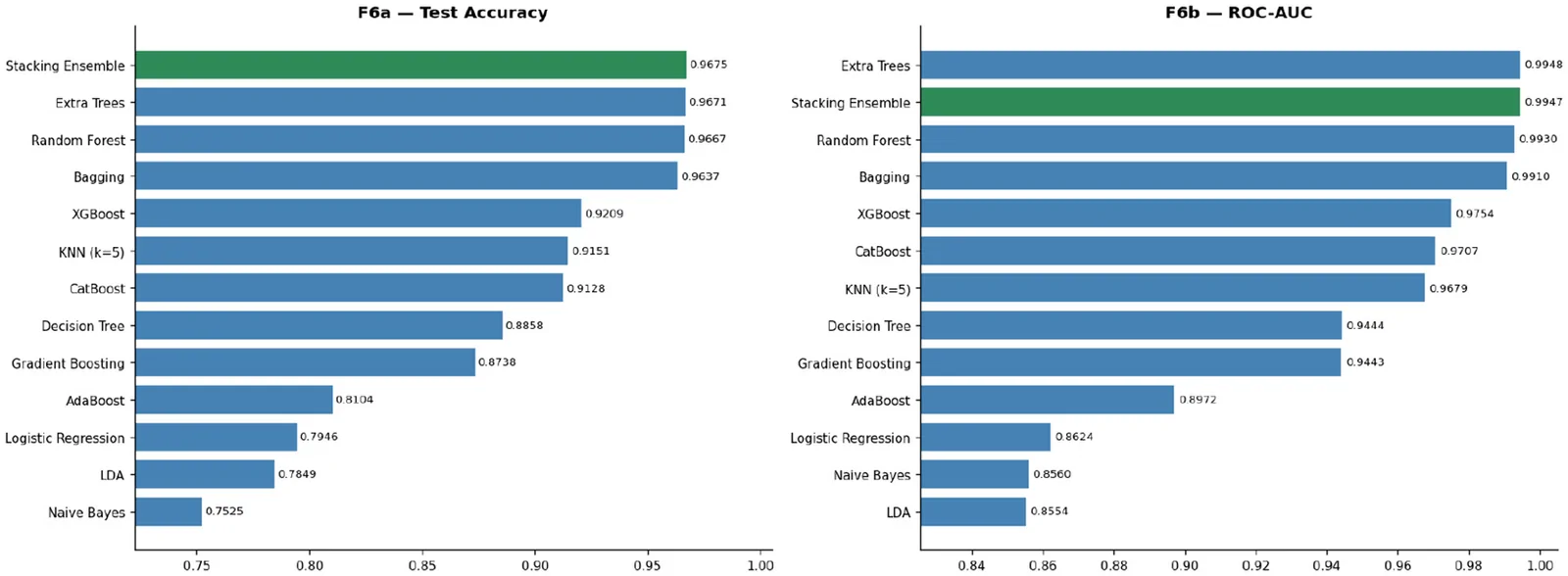
Final accuracy and ROC-AUC ranking of all 13 models. The Stacking Ensemble is the top-ranked model by accuracy (0.9675); Extra Trees is the closest individual baseline (accuracy 0.9671, AUC 0.9948).

**Figure 23:**
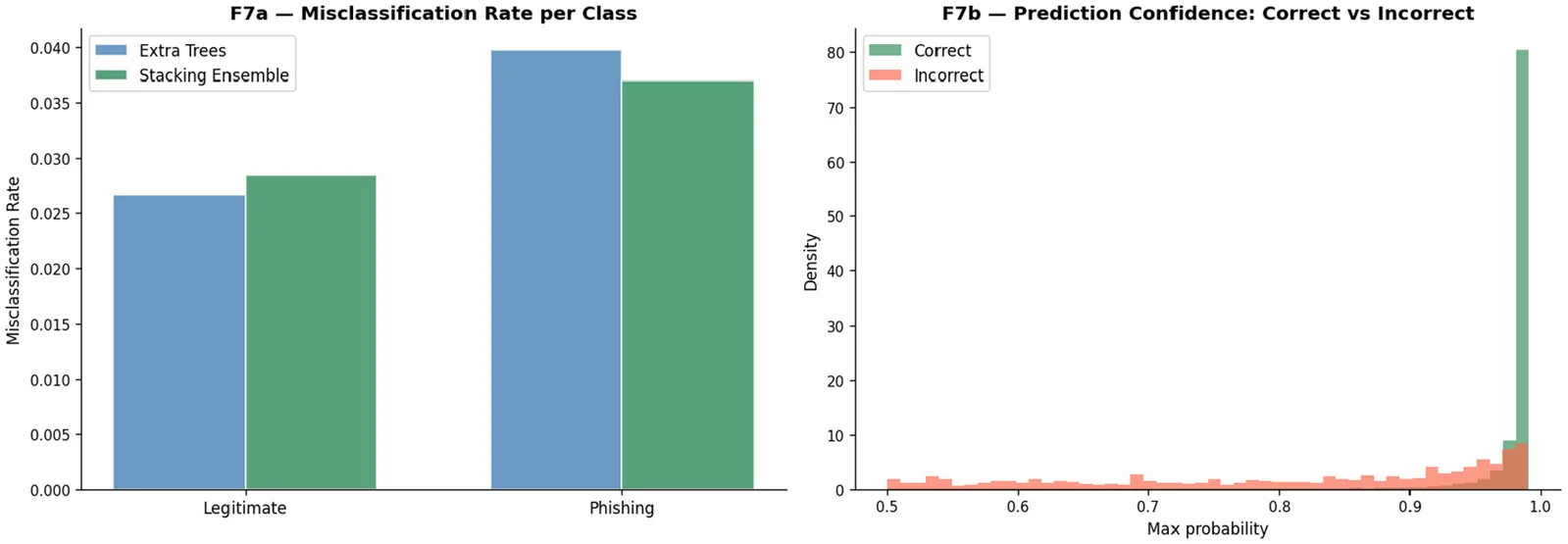
Misclassification analysis for the Stacking Ensemble. **(Left)** Per-class misclassification rates vs. Extra Trees. **(Right)** Prediction confidence distribution showing that misclassified instances are concentrated near the decision boundary ($\hat{p}\approx 0.5$).

### 4.5 Statistical validation

Table 6 presents the complete pairwise consistency results between the Stacking Ensemble and each baseline model. Given the resolution limits of five folds set out in Section 3.9, the table is to be read as a record of directional consistency, not of effect magnitudes.

#### 4.5.1 Friedman test

The Friedman omnibus test yielded $\chi_{F}^{2}=59.84$ with *p* < 0.0001, providing highly significant evidence (α = 0.05) that at least one model performs significantly differently from the others across the five-fold CV distributions (Figure 24). This result justifies all subsequent pairwise *post-hoc* comparisons.

**Figure 24:**
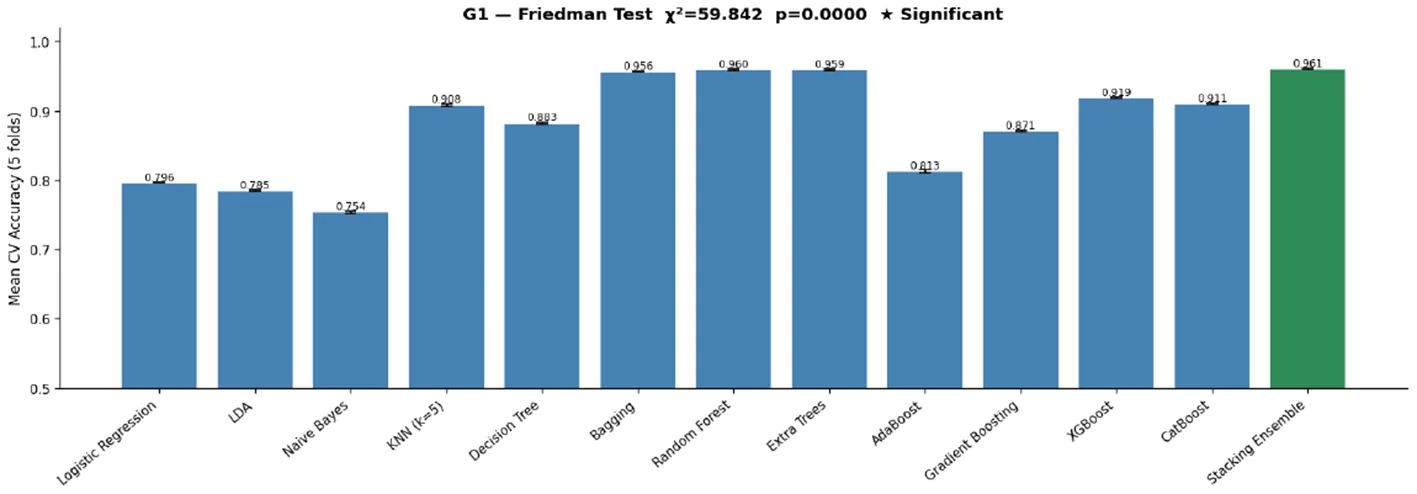
Friedman omnibus test result ($\chi_{F}^{2}=59.84$, *p* < 0.0001), confirming that at least one classifier performs significantly differently from the others and justifying all subsequent pairwise *post-hoc* comparisons.

#### 4.5.2 Wilcoxon signed-rank test

All 12 pairwise Wilcoxon signed-rank tests between the Stacking Ensemble and each baseline model yielded the floor value *p* = 0.0313 (Figure 25). As explained in the methodological note in Section 3.9, this is the minimum attainable *p*-value for *b* = 5 folds and indicates only that the ensemble never lost a fold; it does not measure the magnitude of superiority, which is instead conveyed by Cohen's *d* and by the fold-level CV distributions in Figure 16. The operationally meaningful reading is that, across 12 heterogeneous baselines, the ensemble was never out-performed on any fold—a consistency property that translates directly into deployment confidence, since a detector that is robust to the particular train/validation partition is less likely to regress when retrained on refreshed data.

**Figure 25:**
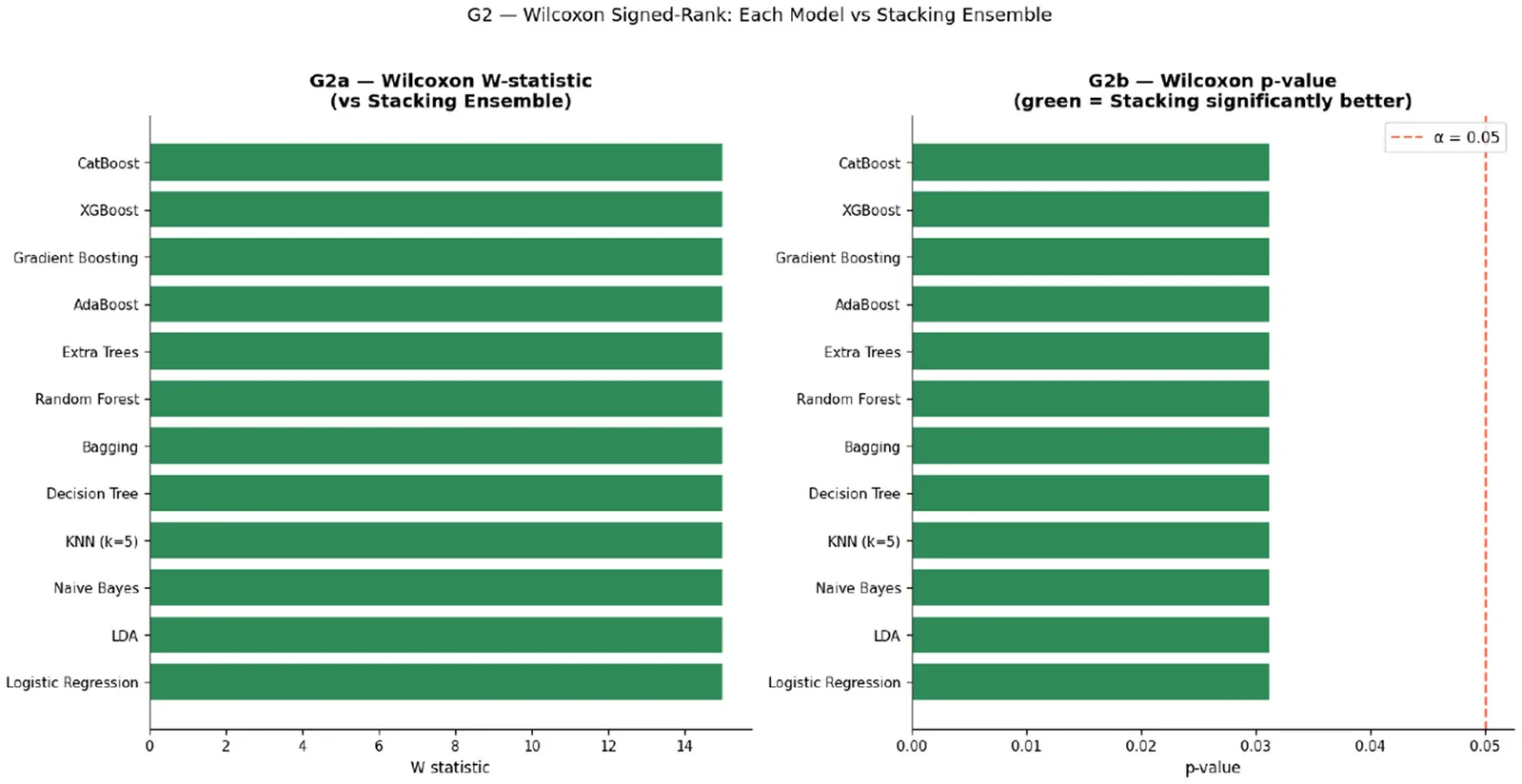
Wilcoxon signed-rank test results for pairwise comparisons between the Stacking Ensemble and each of the 12 baseline classifiers. All 12 comparisons yield *p* = 0.0313 (the minimum achievable *p*-value for *b* = 5 folds), confirming consistent directional superiority across all CV folds.

#### 4.5.3 Paired *t*-test

The paired *t*-test heatmaps (Figure 26) confirmed the Wilcoxon findings across all model pairs. The *t*-statistic heatmap showed consistently positive values in the Stacking Ensemble row (indicating it outperforms all column models), with the largest *t*-statistics observed against linear models (Logistic Regression, LDA, and Naive Bayes) and the smallest against Extra Trees and Random Forest, consistent with the performance rankings in Table 8.

**Figure 26:**
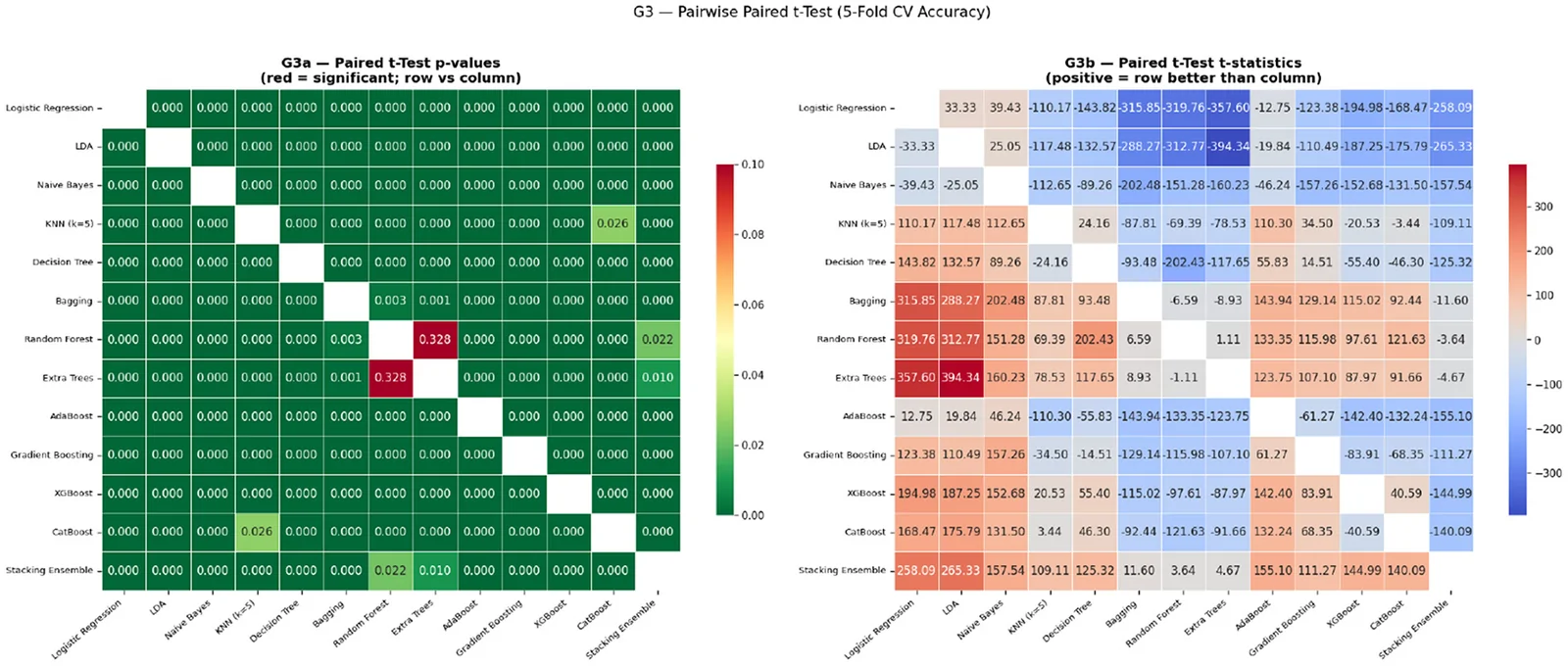
Paired *t*-test heatmaps for all (132) model pairs. Left: *p*-value heatmap. Right: *t*-statistic heatmap (positive values indicate the row model outperforms the column model). The Stacking Ensemble row shows consistently positive *t*-statistics against all 12 baselines.

#### 4.5.4 Effect size analysis

Cohen's *d* values (Figure 27) must be read against the degenerate-variance caveat established in Section 3.9. Ten of the 12 pairwise values—including the extreme *d* = 150.33 against Logistic Regression and *d* = 129.72 against LDA—arise from pooled fold-level standard deviations close to zero and are numerical artifacts of the vanishing denominator in Equation 21; they carry no magnitude information beyond re-confirming that the ensemble won every fold, and we assign them no conventional effect-size label (Table 6). Only two comparisons rest on non-degenerate fold variance and admit conventional interpretation: Random Forest (*d* = 0.73, Medium) and Extra Trees (*d* = 1.03, Large). These are the interpretively meaningful results: despite near-identical test-set accuracy, the Stacking Ensemble's fold-level accuracy distribution is shifted above those of its two closest competitors by roughly one pooled standard deviation, indicating superior robustness to the data partition. Even these two values inherit the caveats of the five-fold, single-seed design (Sections 3.9 and 3.11) and should be regarded as indicative rather than definitive.

**Figure 27:**
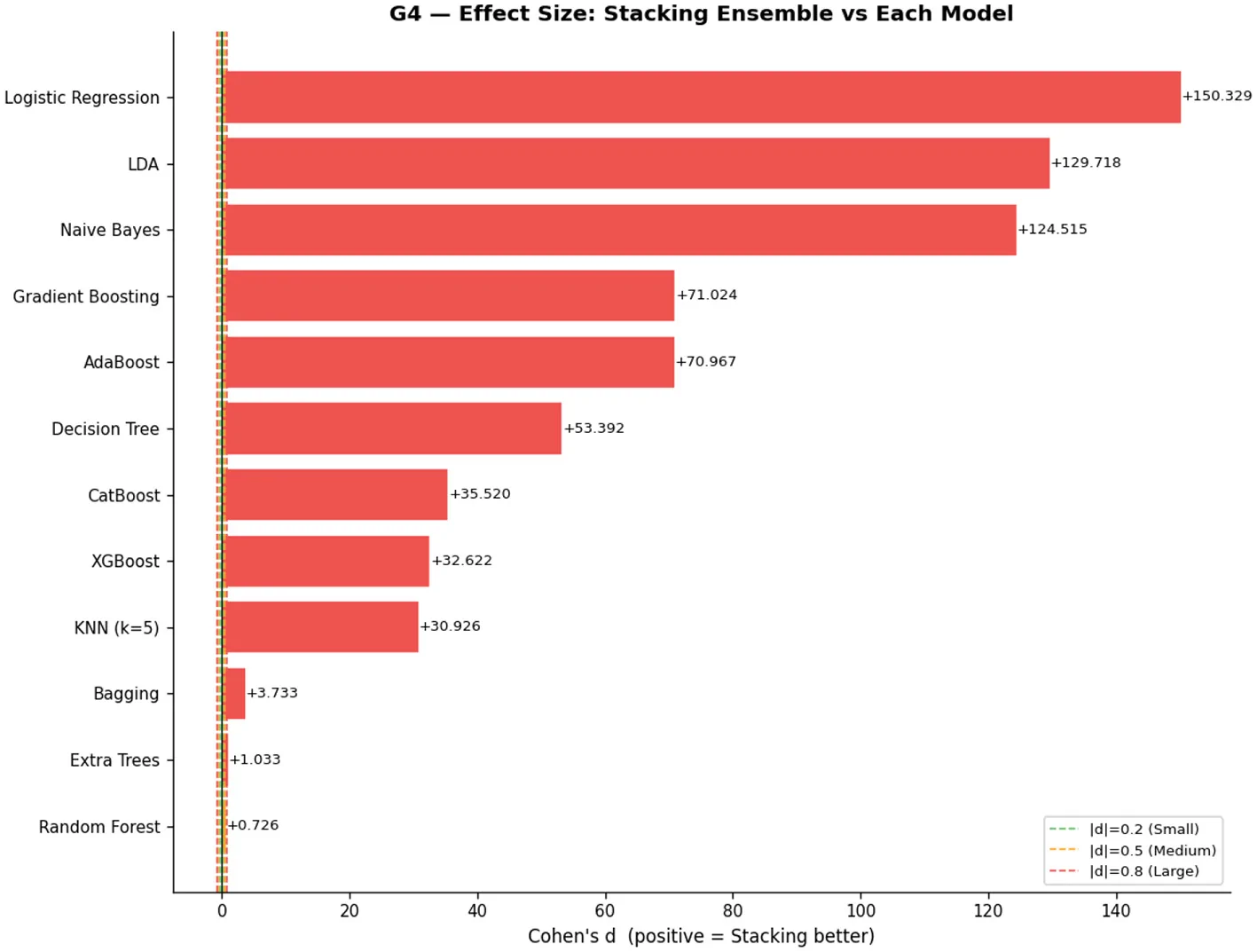
Cohen's *d* effect size for pairwise comparisons between the Stacking Ensemble and each baseline model. Bars are color-coded by magnitude category. Large effect sizes (some inflated by near-zero variance) are observed against 11 of 12 baselines; Medium against Random Forest (*d* = 0.73).

### 4.6 SHAP explainability and feature importance

#### 4.6.1 Ensemble-level SHAP attribution

Figure 28 reports the primary explanation of this study: mean absolute SHAP attributions of the *full stacking ensemble's* phishing-class probability, computed with the model-agnostic explainer described in Section 3.10 on a stratified subsample of the held-out test set. The ensemble's decision function is dominated by subdomain structure—average_subdomain_length and number_of_subdomains occupy the top two ranks—followed by url_length, number_of_dots_in_url, domain_length, and number_of_digits_in_url, with the entropy measures (entropy_of_domain, entropy_of_url) contributing at moderate rank.

**Figure 28:**
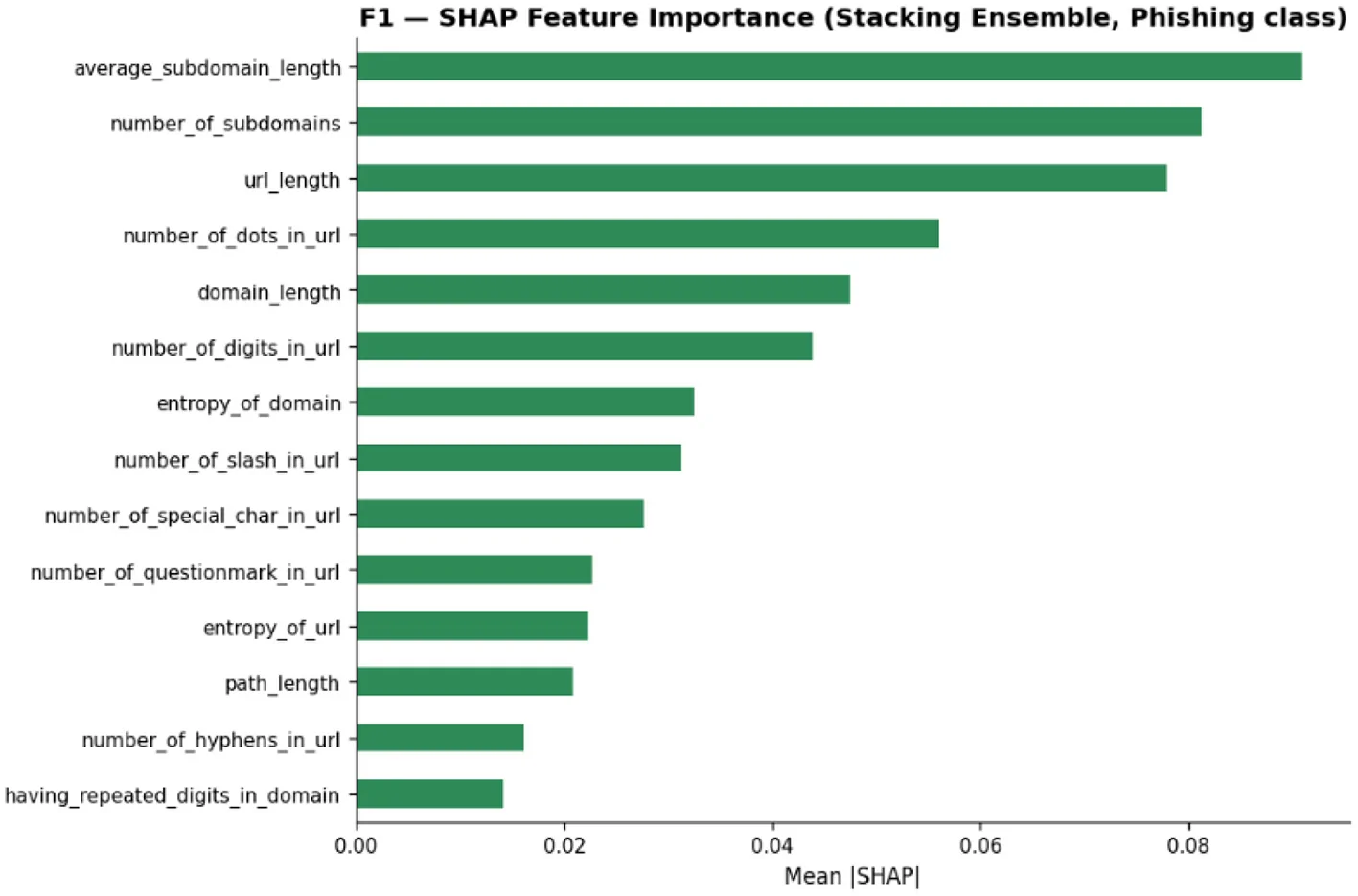
Ensemble-level SHAP feature importance for the Phishing class, computed with a model-agnostic SHAP explainer applied to the full stacking ensemble's positive-class probability on a stratified subsample of the held-out test set, ranked by mean absolute SHAP value. average_subdomain_length and number_of_subdomains are the dominant drivers of the deployed ensemble's phishing score, followed by url_length and dot-count structural features.

The corresponding beeswarm plot (Figure 29) resolves directionality at the instance level. High values of url_length, number_of_dots_in_url, number_of_digits_in_url, and the entropy features (red points) sit at positive SHAP values, pushing the ensemble toward a Phishing verdict, while the subdomain features exhibit a more nuanced pattern in which a dense mass of low-to-moderate values anchors near zero and pronounced deviations in subdomain structure shift predictions in either direction—consistent with subdomain statistics acting as a contextual signal that separates brand-impersonation nesting from the flat subdomain profiles of typical legitimate URLs.

**Figure 29:**
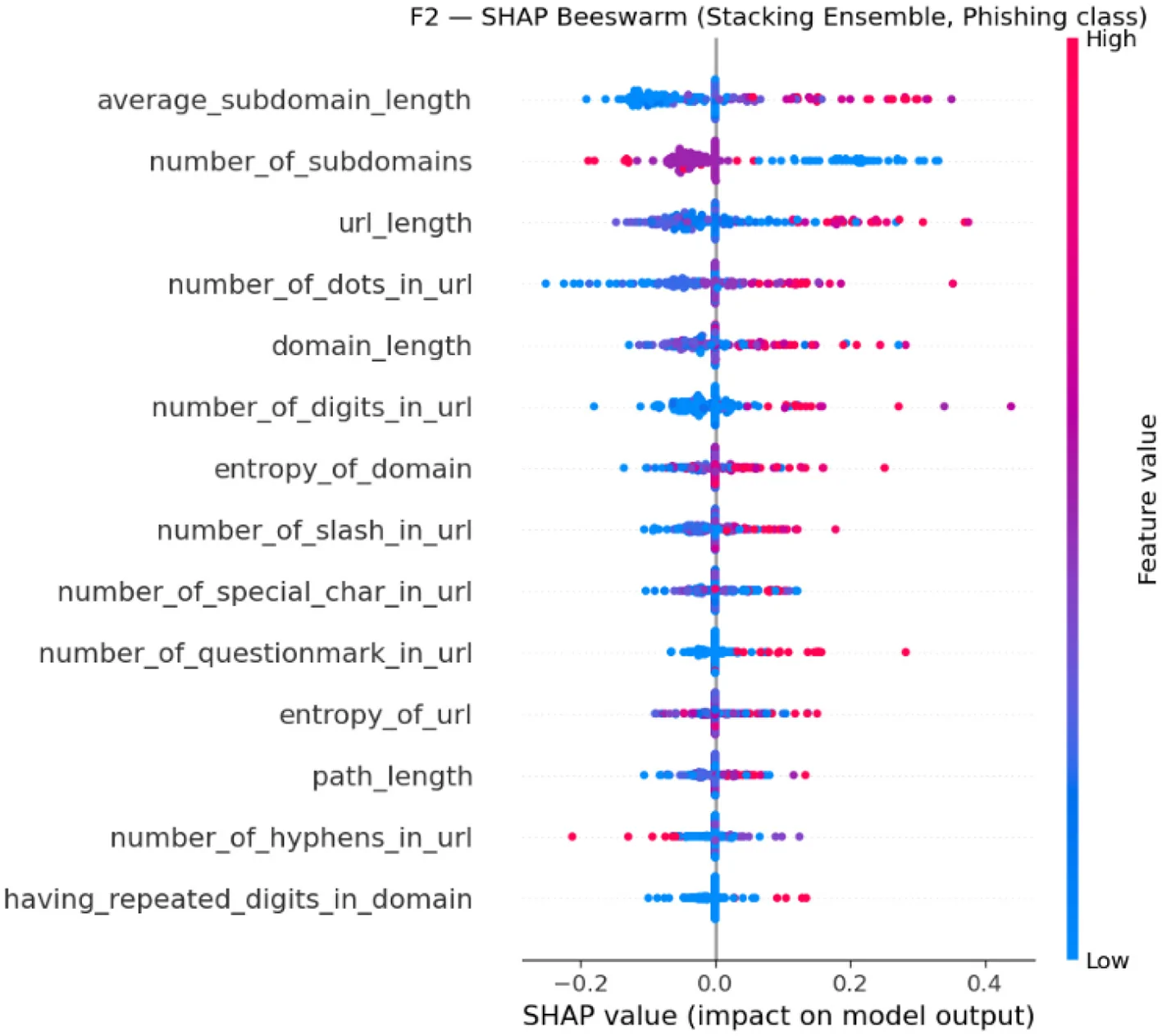
Ensemble-level SHAP beeswarm plot for the Phishing class of the full stacking ensemble on a stratified test-set subsample. Each point is one URL; color encodes feature value (red = high, blue = low) and horizontal position the signed impact on the ensemble's phishing probability. High URL length, dot counts, digit counts, and entropy consistently push predictions toward Phishing.

#### 4.6.2 Base-learner corroboration: global feature importance

As detailed in Section 3.10, exact TreeSHAP attributions were additionally computed on the Optuna-tuned XGBoost base learner over the full held-out test set, serving as a high-resolution cross-check on the sampling-based ensemble-level analysis. The two levels agree closely: XGBoost identifies URL length and structural complexity features as the primary determinants of phishing classification on the test set (Figure 30), the same feature family that dominates the ensemble-level ranking in Figure 28, while URL entropy ranked highest on the training-set probe model (Figure 9). The stability of this feature family across the probe model, the dominant base learner, and the full ensemble indicates that the reported importances are properties of the underlying discriminative structure rather than artifacts of any single model or explanation method. Table 13 reports the top ten features by mean absolute SHAP value on the test set for the XGBoost base learner.

**Figure 30:**
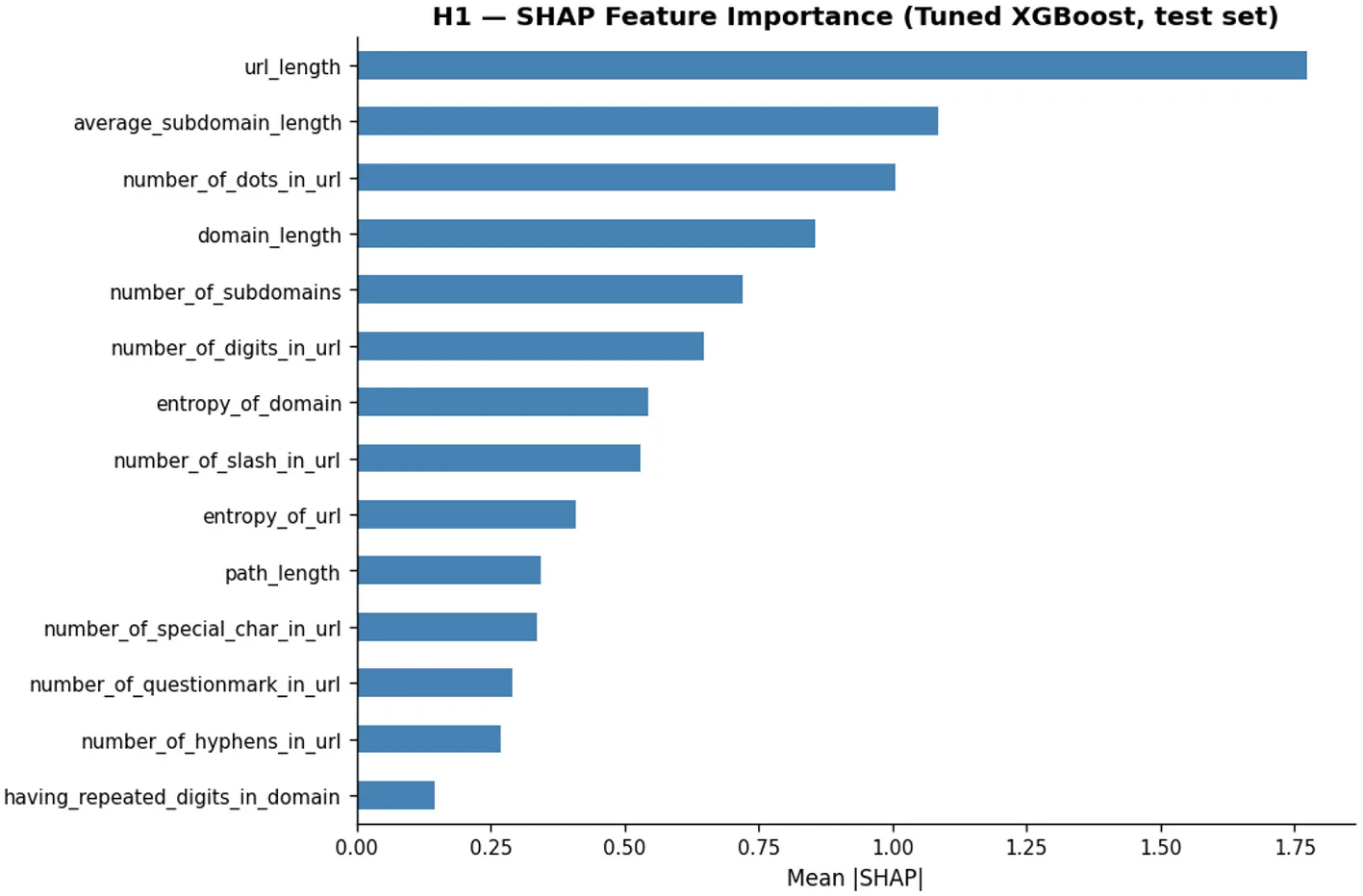
Global SHAP feature importance for the final Optuna-tuned XGBoost base learner on the held-out test set, ranked by mean absolute SHAP value Ī_*j*_. url_length is the dominant predictor on the test set, followed by structural complexity features.

**Table 13:** Top 10 features by mean absolute SHAP value (Optuna-tuned XGBoost base learner, test set).

Feature	Mean |φ_*j*_|	Direction
url_length	Highest	Phishing ↑
average_subdomain_length	High	Phishing ↑
number_of_dots_in_url	High	Phishing ↑
domain_length	Moderate	Phishing ↑
number_of_subdomains	Moderate	Phishing ↑
number_of_digits_in_url	Moderate	Phishing ↑
entropy_of_domain	Moderate	Mixed
number_of_slash_in_url	Moderate	Phishing ↑
entropy_of_url	Lower	Phishing ↑
path_length	Lower	Phishing ↑

Direction indicates the class associated with higher feature values.

URL length and structural complexity features (subdomain length, dot count) ranked as the most influential predictors on the test set, reflecting the tendency of phishing URLs to embed legitimate domain names as path components within longer malicious URLs (Sahingoz et al., 2019). URL entropy, while ranked lower on the test-set model, is consistently the top feature on the training-set probe model, confirming its established role in phishing detection (Le et al., 2018).

#### 4.6.3 Base-learner beeswarm analysis

The XGBoost SHAP beeswarm plot (Figure 31) provided deeper directional insight, and the class-level attribution for the Phishing class (Figure 32) confirmed the same dominant predictors, in agreement with the ensemble-level directional pattern of Figure 29. Higher values of url_length, average_subdomain_length, and number_of_dots_in_url consistently pushed predictions toward Phishing (positive SHAP values), while the presence of a URL path (having_path) was associated with Legitimate classification (negative SHAP values). This is consistent with the observation that phishing URLs frequently omit meaningful path structures, relying instead on query parameters and fragments for payload delivery (Jain and Gupta, 2019).

**Figure 31:**
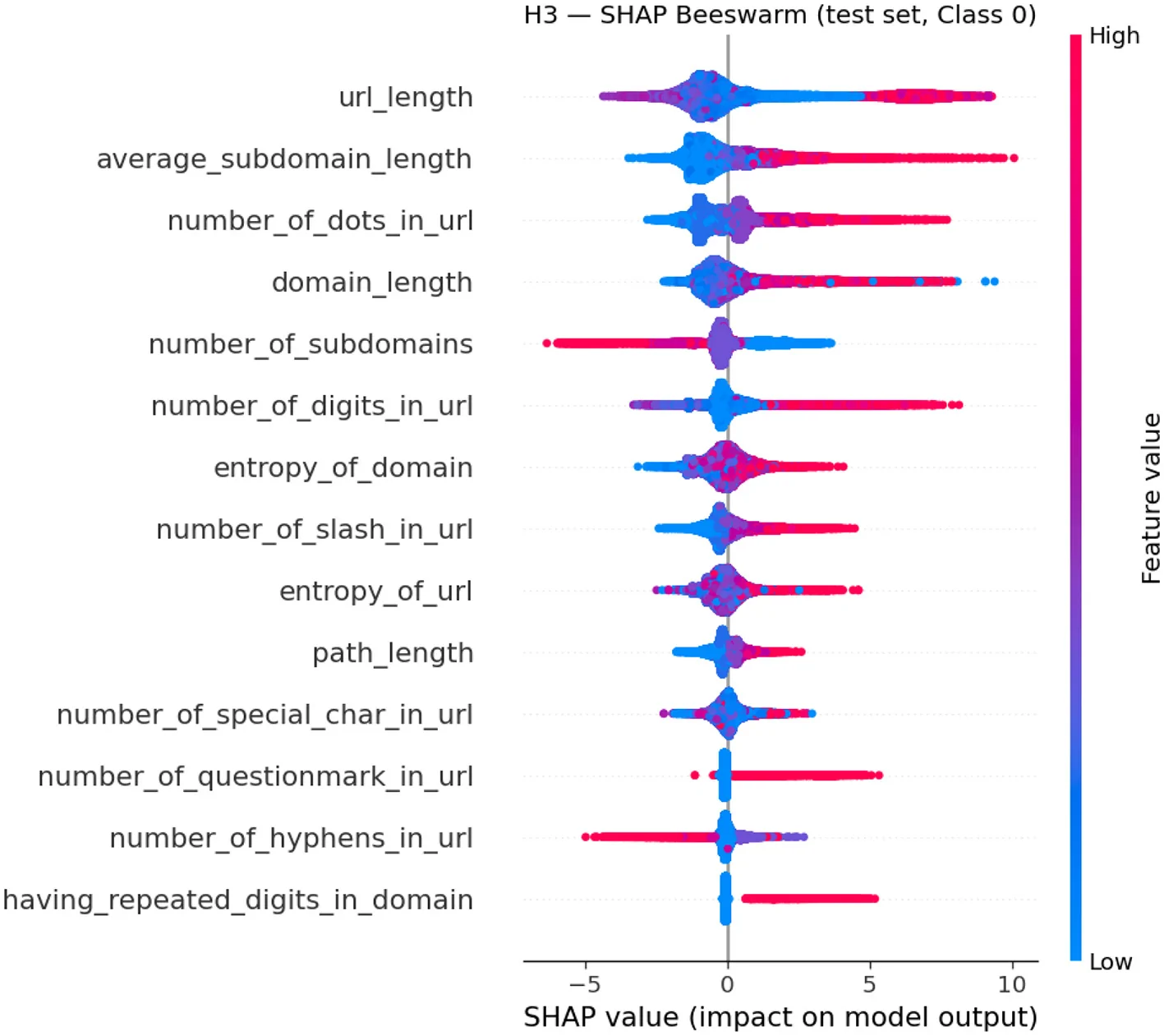
SHAP beeswarm plot for the final Optuna-tuned XGBoost base learner on the test set. Non-linear feature–prediction relationships are visible, enabling security analysts to audit and justify individual URL threat detection decisions at the level of the dominant base model.

**Figure 32:**
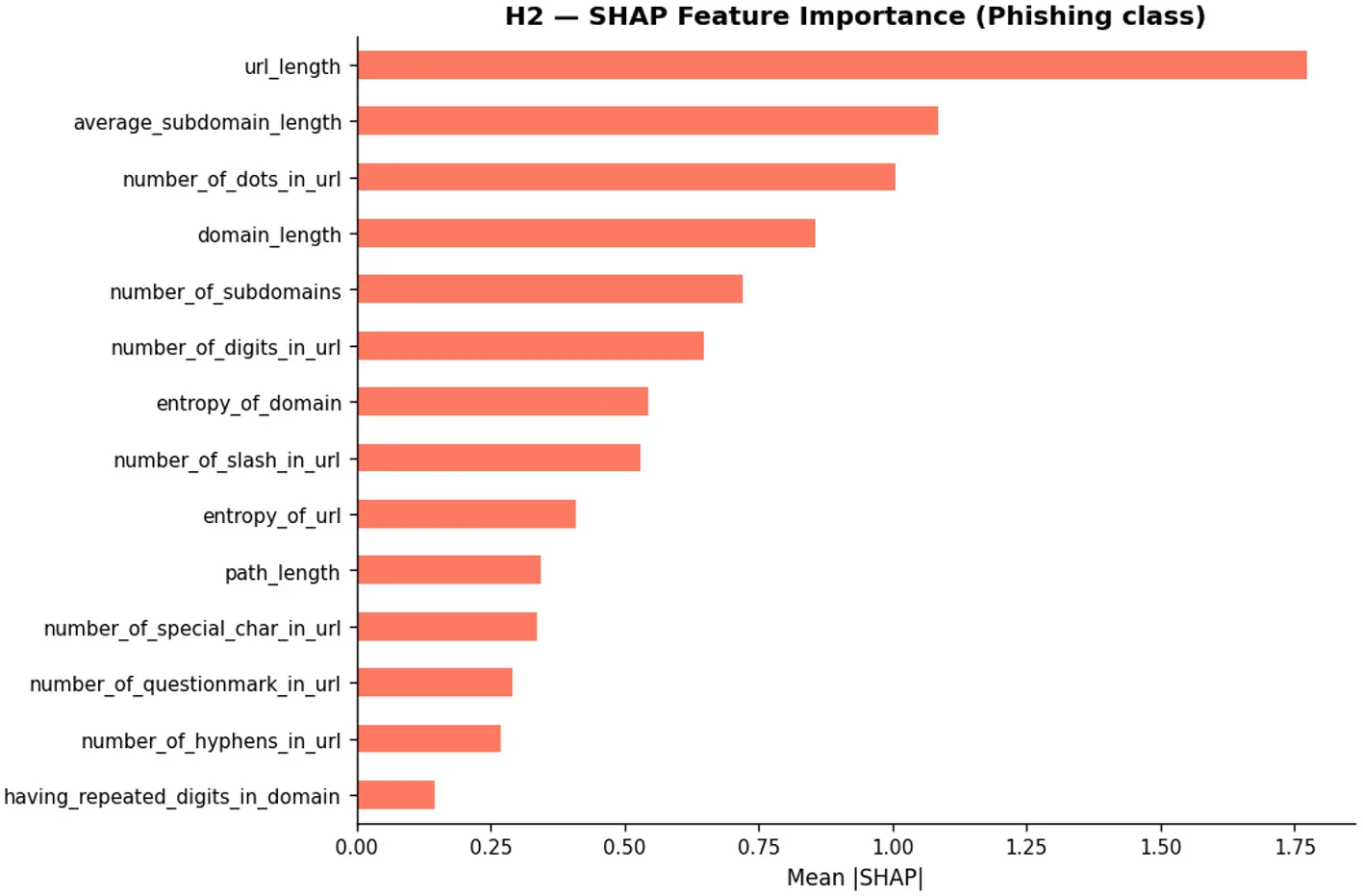
Class-level SHAP feature importance for the Phishing class (Class 1) computed on the Optuna-tuned XGBoost base learner, showing features ranked by their mean absolute SHAP contribution on phishing-predicted samples.

#### 4.6.4 Implications for organizational risk management

The SHAP findings carry operational implications for cybersecurity risk management, subject to an important scope restriction: because all evidence in this study derives from a single benchmark corpus (Section 4.11), the feature rankings support *which signal families* a detection policy should monitor, but not universal numeric cut-offs. The dominance of structural complexity features suggests that URL-based phishing detection policies should prioritize (i) entropy-based screening of URL and domain strings, (ii) heightened scrutiny of deep subdomain nesting, and (iii) secondary inspection of unusually long URLs. Illustrative operating points observed on this corpus—for example, elevated risk beyond roughly three subdomain levels or 150 characters of URL length—are *corpus-specific descriptive statistics, not validated deployment thresholds*: any organization adopting these signals must re-derive the cut-offs from its own traffic distribution, validate them against its own false-positive budget, and re-estimate them after the external validation protocol of Section 4.11 has been carried out. With that qualification, the SHAP-derived signal families are consistent with established cybersecurity heuristics (Jain and Gupta, 2019) and provide a principled, model-informed starting point for tuning organizational security policies and perimeter controls.

#### 4.6.5 Why these features discriminate, in security terms

The prominence of entropy_of_url has a concrete security explanation rather than a purely statistical one. Phishing infrastructure is frequently generated at scale—through domain-generation algorithms, randomized session or tracking tokens, base64- or percent-encoded payloads, and machine-minted subdomains on bulk or compromised hosting—all of which raise the Shannon entropy of the character stream relative to the human-readable, brand-aligned URLs typical of legitimate services. High URL entropy is therefore a proxy for “machine-minted at scale,” which is why it separates the classes so cleanly. The weight assigned to subdomain depth and average_subdomain_length reflects a distinct tactic: attackers nest a trusted brand name in a deep subdomain chain (e.g., paypal.secure-login.account-verify.example.tld) to exploit the fact that users read left-to-right and stop at the first familiar token. Excessive nesting is thus an impersonation signal, not merely a length artifact. The contribution of overall URL length captures the common pattern of padding a malicious destination with a legitimate-looking path or redirect chain.

#### 4.6.6 Adversarial evasion and its limits

Because these features are observable to attackers, they are also manipulable: an adversary can deliberately shorten URLs, lower entropy by using dictionary words instead of random tokens, or flatten the subdomain structure to mimic benign URLs. Lexical-only detection is therefore best treated as one layer of a defense-in-depth strategy rather than a standalone control, and the model should be retrained periodically on fresh campaigns to track distribution drift. Crucially, evasion is not free for the attacker: suppressing entropy and structural complexity constrains the very automation and obfuscation that make large-scale phishing economical, so even an evaded classifier raises the attacker's cost. This motivates pairing lexical scoring with host-based and content-based signals, and monitoring the SHAP distribution over time as an early indicator of adversarial adaptation.

#### 4.6.7 Use of SHAP outputs in a SOC

For a security operations center, the value of per-instance SHAP attributions is operational rather than merely descriptive. Analyst-facing tooling can surface the top contributing features for each flagged URL, allowing a tier-1 analyst to triage an alert in seconds (“flagged on high entropy and four-level subdomain nesting”) instead of inspecting the raw URL unaided, directly reducing alert fatigue and mean-time-to-triage. The same attributions can enrich SIEM events, justify automated block decisions in audit logs, and feed back into blocklist and web-application-firewall rule tuning by exposing which structural patterns currently dominate detections. Aggregated over a detection window, shifts in the global SHAP ranking carry threat-intelligence value by revealing when attacker tradecraft is migrating from one structural signature to another.

### 4.7 Computational cost and runtime complexity

Deployment feasibility depends not only on accuracy but on training and inference cost. Table 14 summarizes the asymptotic time complexity of each model family and of the stacking ensemble, where *n* is the number of training samples, *d* the number of selected features (*d* = 24 after SHAP selection), *T* the number of trees (or boosting iterations), and *M* = 4 the number of base learners in the ensemble. These bounds are architecture-independent and therefore portable across hardware, in contrast to wall-clock measurements, which depend on the specific CPU/GPU, library build, and parallelism available.

**Table 14:** Asymptotic training and per-sample inference time complexity.

Model family	Training	Inference (per URL)
Logistic Regression/LDA	O(nd)	O(d)
Naive Bayes	O(nd)	O(d)
KNN (*k* = 5)	O(1) (lazy)	O(nd)
Decision Tree	O(ndlogn)	O(depth)
Random Forest/Extra Trees/Bagging	O(Tndlogn)	O(T·depth)
AdaBoost/Gradient Boosting/XGBoost/CatBoost	O(Tnd)	O(T·depth)
Stacking Ensemble	$O\left(\underset{m}{\sum }C_{m}^{\text{train}}+nM\right)$	$O\left(\underset{m}{\sum }C_{m}^{\text{infer}}+M\right)$

*n*, training samples; *d*, features; *T*, trees/iterations; *L*, leaves; *M*, number of base learners. The stacking ensemble's cost is the sum of its base learners' costs plus the out-of-fold cross-validation overhead and a lightweight meta-learner.

Two practical observations follow. First, the instance-based KNN baseline is the only model whose inference cost grows with the training-set size [O(nd) per query], making it the least suitable for high-throughput URL screening despite its competitive accuracy; all tree-based and linear models have inference cost independent of *n*. Second, the stacking ensemble is necessarily more expensive than any single base learner: at training time it fits all four base learners under a five-fold out-of-fold scheme to generate the meta-features, so its training cost is approximately the sum of the base learners' costs plus a five-fold cross-validation factor, and at inference time it evaluates all four base learners followed by a constant-time logistic-regression meta-step. In exchange for this overhead it delivers the improved calibration and fold-level stability reported in Section 4.5. Because all four base learners are evaluated independently, ensemble inference is trivially parallelizable across cores.

For the operating point used here (*d* = 24 features, tree depths in the single digits, and a few hundred trees per ensemble member), inference is dominated by a small number of shallow tree traversals and a four-dimensional logistic-regression evaluation, which is why the framework runs on commodity CPU hardware without GPU dependency—in contrast to the character-level CNN and CNN+BiLSTM baselines discussed in Section 5, whose inference requires dense sequence-model evaluation. We deliberately refrain from quoting precise wall-clock latency figures here, as these are sensitive to the deployment environment; the released run logs (see the Section Data Availability Statement) record the measured training and inference times on the experimental platform, and practitioners should benchmark on their own target hardware before committing to a throughput service-level objective.

### 4.8 Contextualization with prior work

Table 15 lists representative prior studies on URL-based phishing detection. We emphasize at the outset that this table is *not* a head-to-head benchmark and the reported accuracies are *not* directly comparable: the listed studies use different datasets (of widely varying size, age, and phishing-campaign composition), different feature spaces (raw character sequences vs. engineered structural features vs. deep-learned representations), and different evaluation protocols (single train/test split vs. *k*-fold CV; unstated vs. documented leakage controls). A 0.98 reported on one dataset and a 0.97 reported on another differ along at least three axes besides model quality, and the literature does not provide a common benchmark on which all of these methods have been re-evaluated. We therefore present this table strictly as *contextual positioning*, illustrating where the proposed framework sits within the broader landscape of URL phishing detectors at a high level, and we draw no claim of superiority or inferiority from the per-row accuracy figures.

**Table 15:** Contextual positioning of the proposed framework against representative prior studies on URL-based phishing detection.

References	Method	Reported acc.	AUC	Features	Dataset/protocol
Sahingoz et al. (2019)	Random Forest (NLP)	0.9798	–	22	73,575 URLs (own corpus); single split
Vrbančič et al. (2020)	Gradient Boosting	0.9730	–	111	Five different phishing datasets
Yang et al. (2019)	CNN (character)	0.9812	–	Raw URL	Own corpus; deep-learning protocol
Feng et al. (2020)	CNN+BiLSTM	0.9850	–	Raw URL	Own corpus; deep-learning protocol
**Proposed (Stacking Ensemble)**	Stacking + SHAP	**0.9675**	**0.9947**	**24**	247,950 URLs; leakage-free five-fold CV + held-out test

Accuracies are reported as published by each study on its own dataset and protocol; they are *not* directly comparable across rows. Bold values indicate the highest reported accuracy within the table. Note that accuracies reported across rows are not directly comparable, as each study used a different dataset and evaluation protocol.

Beyond the headline accuracy figures, the proposed framework offers four methodological properties that are absent or under-documented in the compared studies and that are arguably more important than raw accuracy for production deployment: (i) an explicitly leakage-free pipeline in which every preprocessing, feature-selection, and hyperparameter-tuning step is fit exclusively on *D*_train_; (ii) multi-level SHAP explainability spanning global, class-level, and instance-level attribution; (iii) a four-layer statistical validation framework with omnibus, pairwise non-parametric, pairwise parametric, and effect-size analyses; and (iv) millisecond inference on commodity hardware without GPU dependency, in contrast to the CNN and CNN+BiLSTM baselines. These properties—not the second-decimal accuracy figure—constitute the framework's primary contribution relative to prior work; a direct benchmark on a shared dataset (e.g., the Vrbančič et al. corpus) is an important avenue for future work.

### 4.9 Implications for business decision-making and organizational resilience

The experimental findings carry several concrete implications for AI-driven cybersecurity governance in organizational contexts.

#### 4.9.1 Risk-calibrated deployment

The strong calibration of the Stacking Ensemble's probability outputs (lowest Brier score among all models; Table 12) makes its scores suitable, in principle, for tiered response frameworks. As an *illustrative template*, URLs assigned $\hat{p}>0.9$ could be automatically blocked, those in the [0.6, 0.9] range escalated for human review, and those below 0.6 permitted with logging. We emphasize that these tier boundaries are illustrative only: calibration measured on one benchmark does not guarantee calibration on an organization's live traffic, so any deployment must first complete the external-validation and recalibration protocol of Section 4.11 and set tier boundaries from probabilities recalibrated on in-environment data. With that precondition, the tiered approach supports the risk-quantification function of AI-driven organizational resilience (Gordon and Loeb, 2002).

#### 4.9.2 Threshold-aware policy design

The threshold analysis results demonstrate that security teams can shift τ to achieve their desired precision-recall operating point without retraining the model. Organizations operating in high-stakes financial or healthcare environments may prefer τ < 0.5 to prioritize recall, accepting a higher false-positive rate as the cost of enhanced protection (Elkan, 2001); as with the tier boundaries above, the specific operating point should be selected on validated, in-environment score distributions rather than transferred directly from this benchmark.

#### 4.9.3 Explainability for stakeholder communication

The SHAP global importance rankings provide security leadership with a concise, evidence-based narrative for board-level risk reporting (Arrieta et al., 2020).

#### 4.9.4 Statistical rigor for governance compliance

The four-layer statistical validation framework provides the evidentiary standard required for AI governance compliance under frameworks such as the NIST AI Risk Management Framework (National Institute of Standards and Technology, 2018) and emerging EU AI Act requirements.

### 4.10 Limitations and external validity

The metrics reported in this study are obtained on a single public benchmark through a leakage-controlled internal split, and should be read as *in-distribution and provisional* with respect to external generalization. No cross-dataset or temporal out-of-distribution evaluation was performed; the headline figures therefore characterize performance on the Phishing URL Detection Dataset and do not, by themselves, establish that the framework transfers to URL corpora with different feature distributions, phishing-campaign typologies, collection periods, or linguistic and geographic composition. We make this boundary explicit to avoid over-generalizing from a single benchmark.

Two methodological limitations compound the single-corpus scope. First, the statistical protocol operates on only five paired observations per comparison: the Wilcoxon test is pinned at its combinatorial floor, the paired *t*-test has four degrees of freedom, and most Cohen's *d* values are rendered uninterpretable by degenerate fold variance (Section 4.5), so the analysis establishes directional consistency rather than calibrated effect magnitudes; repeated cross-validation or corrected resampled tests over more folds would be required for the latter. Second, all stochastic operations share the single seed 42, so the reported fold-level variance captures data-partition effects only and excludes algorithmic stochasticity (Section 3.11); multi-seed replication is required to characterize total run-to-run variability, and both extensions are identified as immediate follow-on work.

Several specific threats to external validity warrant emphasis. Temporal drift means that phishing tradecraft evolves, so a model trained on one collection window may degrade on URLs gathered later. The engineered feature set is specific to this corpus, and another dataset may expose different or differently distributed attributes. Sampling and labeling conventions differ across public phishing datasets, which can shift the apparent decision boundary. Finally, the adversarial non-stationarity discussed in Section 4.7 implies that static benchmark accuracy may overstate field performance against adaptive attackers.

Rather than claim external validity we did not test, we specify the protocol that would establish it as the immediate next step:

1. *Cross-dataset transfer*—train on the present corpus and evaluate, without any refitting or re-selection, on one or more independent public phishing datasets, reporting the in- to out-of-distribution performance gap;
2. *Temporal robustness*—partition URLs by collection date, train on the earlier period, and test on the later period to quantify drift; and
3. *Recalibration analysis*—measure how much of any degradation is recovered by threshold recalibration alone vs. full retraining.

We regard this evaluation as a prerequisite for any deployment-level claim of generalization, and we report the present results strictly as a rigorous, reproducible in-distribution baseline on which that external validation can build.

## 5 Conclusion

This paper has presented and validated a comprehensive AI-driven cybersecurity risk analytics framework for phishing URL detection that simultaneously addresses the three persistent limitations of existing approaches: limited benchmarking breadth, absence of model interpretability, and insufficient statistical rigor in performance validation. The proposed framework integrates 12 analytical stages into a strictly leakage-free pipeline, evaluated on a large-scale benchmark of 247,950 URL records with 41 structural and entropy-based features.

The key findings of this study can be summarized as follows. First, SHAP-driven feature selection reduced the original 41-feature space by 41.5% to 24 features while retaining 95% of the cumulative predictive signal. Second, the 12-model benchmarking study revealed three distinct performance tiers, with tree-based ensemble methods substantially outperforming linear and probabilistic classifiers, confirming that the Legitimate/Phishing decision boundary is fundamentally non-linear in URL feature space. Third, Bayesian hyperparameter optimization via Optuna TPE sampling improved XGBoost cross-validated accuracy by 1.90 percentage points and CatBoost by 1.53 percentage points in 40 trials. Fourth, the Bayesian-optimized stacking ensemble and Extra Trees emerged as co-leaders, with the stacking ensemble attaining the highest accuracy (96.75%), F1-score (96.74%), MCC (0.9348), and fold-level stability, and Extra Trees retaining a marginal, statistically uncharacterized ROC-AUC edge (0.9948 vs. 0.9947) that lies within fold-level noise; the ensemble delivers per-class precision and recall of 0.97 across both Legitimate and Phishing categories, and its primary practical advantage over Extra Trees is its probability calibration—the lowest Brier score of all 13 models (0.0246 vs. 0.0249)—and fold-level consistency rather than raw ranking-metric accuracy. Fifth, the four-layer statistical consistency analysis—Friedman omnibus test ($\chi_{F}^{2}=59.84$, *p* < 0.0001), Wilcoxon signed-rank tests (*p* = 0.0313 for all 12 pairwise comparisons, the minimum achievable *p*-value for *b* = 5 folds and therefore an indicator of directional consistency rather than magnitude), pairwise paired *t*-tests, and Cohen's *d* effect sizes (interpretable only for the two non-degenerate-variance comparisons)—confirmed that the stacking ensemble's advantage is directionally consistent across all five cross-validation folds, within the resolution limits of that design. Sixth, *post-hoc* SHAP explainability—applied at the ensemble level via a model-agnostic explainer on the full stacking pipeline and corroborated by exact TreeSHAP on the Optuna-tuned XGBoost base learner—identified subdomain structure, URL length, and URL entropy as the dominant phishing indicators, providing security analysts with concise, model-validated threat intelligence.

Several limitations of the present study warrant acknowledgment. The framework was evaluated on a single benchmark dataset, and generalization to other URL corpora with different feature distributions, language characteristics, or phishing campaign typologies remains to be established; for the same reason, the deployment tiering and screening thresholds discussed in Sections 4.7 and 4.10 are presented as illustrative templates rather than validated operational prescriptions. The five-fold cross-validation procedure provides only five data points for statistical testing, constraining the power of pairwise significance tests, producing a Wilcoxon *p*-value floor of 0.0313, and rendering most Cohen's *d* values uninterpretable as conventional effect sizes; and because all stochastic operations share a single fixed seed, the reported fold-level variance reflects data-partition effects only. The ensemble-level SHAP attribution, while targeting the deployed pipeline directly, is computed with a sampling-based model-agnostic explainer on a test-set subsample and is therefore approximate; its close agreement with the exact TreeSHAP attributions of the dominant XGBoost base learner is the basis on which we treat the reported rankings as reliable. Additionally, the framework evaluates URLs in isolation without incorporating temporal dynamics, network context, or page-content signals.

Future research directions include: (i) adversarial robustness evaluation against URL obfuscation attacks; (ii) real-time streaming URL classification with online learning components; (iii) cross-domain transfer to network intrusion detection, malware classification, and spam filtering; (iv) integration of large language model-based semantic URL analysis; (v) multi-dataset validation across diverse geographic and linguistic URL corpora, together with multi-seed replication and higher-fold repeated cross-validation, to establish the generalizability and total run-to-run variability of the framework's performance and explainability findings; and (vi) direct head-to-head benchmarking against the deep-learning baselines listed in Table 15 on a shared corpus, since the present comparison is necessarily contextual rather than competitive.

## Data Availability

The original contributions presented in the study are included in the article/Supplementary material, further inquiries can be directed to the corresponding author.
